# “Key to the Future”: British American Tobacco and Cigarette Smuggling in China

**DOI:** 10.1371/journal.pmed.0030228

**Published:** 2006-07-18

**Authors:** Kelley Lee, Jeff Collin

**Affiliations:** **1**Centre on Global Change and Health, London School of Hygiene and Tropical Medicine, London, United Kingdom; **2**Centre for International Public Health Policy, School of Health in Social Science, University of Edinburgh, Edinburgh, United Kingdom; University of CaliforniaUnited States of America

## Abstract

**Background:**

Cigarette smuggling is a major public health issue, stimulating increased tobacco consumption and undermining tobacco control measures. China is the ultimate prize among tobacco's emerging markets, and is also believed to have the world's largest cigarette smuggling problem. Previous work has demonstrated the complicity of British American Tobacco (BAT) in this illicit trade within Asia and the former Soviet Union.

**Methods and Findings:**

This paper analyses internal documents of BAT available on site from the Guildford Depository and online from the BAT Document Archive. Documents dating from the early 1900s to 2003 were searched and indexed on a specially designed project database to enable the construction of an historical narrative. Document analysis incorporated several validation techniques within a hermeneutic process. This paper describes the huge scale of this illicit trade in China, amounting to billions of (United States) dollars in sales, and the key supply routes by which it has been conducted. It examines BAT's efforts to optimise earnings by restructuring operations, and controlling the supply chain and pricing of smuggled cigarettes.

**Conclusions:**

Our research shows that smuggling has been strategically critical to BAT's ongoing efforts to penetrate the Chinese market, and to its overall goal to become the leading company within an increasingly global industry. These findings support the need for concerted efforts to strengthen global collaboration to combat cigarette smuggling.

## Editors' Summary

**Background.:**

Smoking tobacco kills about 5 million people around the world every year, and according to the World Health Organization, half of the people who smoke will eventually die as a result. The habit is in decline in most of the world's richer nations, but it is growing in developing countries. Governments can control consumption, and raise substantial tax revenues, by levying high taxes on tobacco. However, this is undermined when tobacco is smuggled into the country and sold illegally. Smuggling makes cigarettes more affordable, thus stimulating consumption, and undermines efforts to reduce smoking rates.

British American Tobacco (BAT) is one of the largest international tobacco companies. Following a legal case in the United States, millions of pages of BAT internal documents (dating back to the early 1900s) have been made public. They are held in a depository in the United Kingdom, and most can now be seen online. Researchers have already undertaken wide-ranging studies of these documents and have drawn conclusions about the way BAT has sought to enter emerging markets throughout the world.

**Why Was This Study Done?:**

Previously published studies have concluded that BAT has been “complicit” in large-scale cigarette smuggling, described in documents by the euphemisms “transit,” “general trade” (“GT”), “duty not paid” (“DNP”), and “free market.” This study focuses on such activities in China, which is believed to have the world's biggest tobacco smuggling problem. China is a key market for BAT and other tobacco companies because of the size of its population, high smoking prevalence, and rapidly growing economy. The development of effective tobacco control policy in China constitutes one of the greatest opportunities to advance global health. The State Tobacco Monopoly Administration is the only agency legally allowed to trade in tobacco in China.

**What Did the Researchers Do and Find?:**

The authors have put together an account of the company's trading in China in the period from 1986 to 1999. They begin by describing the scale on which tobacco was smuggled into China and the main supply routes through which tobacco entered the country. They found evidence that smuggling increased considerably during the period.

The researchers found that BAT's own documents describe the company's efforts to improve its earnings from China by restructuring operations and controlling the supply chain—that is, how cigarettes are manufactured and legally exported, are handled by various “agents” or middlemen, and then are sold illicitly as contraband. The title of this article is taken from one BAT document that states that exports from Hong Kong to China are the “key to the future” of the company's planned growth. The researchers say that this can be taken to mean smuggling, and that BAT's documents indicate that the company benefited from, encouraged, and attempted to control the smuggling of tobacco into China.

**What Do These Findings Mean?:**

BAT has stated publicly that it does not approve of smuggling. However, from the researchers' analysis of the information in the internal papers, they conclude that “the documents demonstrate that contraband has been a hugely profitable and integral part of BAT operations in China over the past two decades.” They estimate that the value of the illicit trade amounts to billions of (United States) dollars and go on to say that “for the Chinese government, this illicit trade undermined restrictions on imports, represented an enormous loss of tax revenue, and stimulated demand for premium brand cigarettes.” The researchers argue that action should be taken internationally to reduce tobacco smuggling.

**Additional Information.:**

Please access these Web sites via the online version of this summary at http://dx.doi.org/10.1371/journal.pmed.0030228.

• Most of the documents used in this paper can be found at the British American Tobacco Documents Archive created by public health researchers (the Web site also contains useful information on tobacco document research)

• The Tobacco Free Initiative, based at the World Health Organization, provides information on the global scale of the tobacco problem, and about efforts to combat the situation

• Many countries have national anti-smoking organizations; the United Kingdom charity Action on Smoking and Health has a useful list of these

• Information about the dangers of smoking to personal health and advice on giving up the habit are available from the Web sites of the American Academy of Family Physicians and NHS Direct (United Kingdom National Health Service)

## Abstract 3

Internal documents from British American Tobacco demonstrate the scale of tobacco smuggling into China and show that this illicit trade has been a critical factor in BAT's expansion in this market.

## Introduction

Cigarette smuggling has emerged as a critical public health issue. As well as being the source of huge losses in government revenues, smuggling makes cigarettes more affordable, thus stimulating consumption and undermining control measures, most notably among youth and low-income consumers [ 1, 2]. The illicit trade in tobacco is served by three broad categories of supply, namely, counterfeiting, bootlegging, and large-scale smuggling. This paper focuses on large-scale smuggling of cigarettes and other tobacco products that circumvent tax regimes, which accounts for the vast majority of cigarettes smuggled globally [ 1]. Recent estimates suggest that the trade in smuggled cigarettes accounts for around 6%–8% of global consumption [ 3] and around one-quarter of total exports [ 4]. The centrality of provisions to combat smuggling within the World Health Organization's Framework Convention on Tobacco Control (FCTC) [ 5] indicate recognition of this worldwide problem.

Tobacco industry documents, released following litigation in the United States, have demonstrated the complicity of British American Tobacco (BAT) in large-scale smuggling [ 6]. Whereas previous work has illustrated the strategic importance of smuggling to BAT's expansion in Asia [ 7] and the former Soviet Union [ 8], this paper offers the first detailed analysis of corporate involvement in contraband to one country. The significance of this work is enhanced by China's status as the world's largest cigarette market, comprising 350 million smokers and one-third of cigarettes smoked [ 9]. So far dominated by domestic producers, China is seen as the “ultimate prize” [ 10] among the world's emerging tobacco markets because of a potentially huge demand for foreign brands.

China has long been recognised as also having “possibly the largest cigarette smuggling problem in the world” [ 11]. Legal imports of foreign cigarettes comprise about 3% of the 1.7 trillion (1.7 × 10 ^12^) stick market. However, up to 50 billion (5 × 10 ^10^) foreign cigarettes were believed to be entering the country illegally each year by the late 1990s [ 11– 13]. In recent years, Chinese government efforts to crack down on smuggling activities, and recover some of the US$1.8 billion in lost tax revenue annually [ 12], have led to the strengthening of the General Administration of Customs, including administrative and criminal penalties for smuggling [ 14]. While there have been criminal prosecutions of some individuals and widely reported media accounts of this illicit trade [ 6, 15], scholarly research on the role of tobacco companies remains limited. Using evidence from internal documents of the tobacco industry, this paper begins by describing the huge scale of this illicit trade and the key supply routes by which it has been conducted. It examines BAT's efforts to optimise earnings by restructuring operations and controlling the supply chain (how cigarettes are manufactured and legally exported, are handled by various “agents” or middlemen, and then sold illicitly as contraband) and the pricing of smuggled cigarettes. Indeed, documents produced in litigation demonstrate that smuggling has been strategically critical to BAT's ongoing efforts to penetrate the Chinese market, the “key to the future” [ 16] in BAT's overall goal to become the leading company within an increasingly global industry.

## Methods

This paper is based on analysis of documents dating between the early 1900s and 2003 from the BAT Depository in Guildford, United Kingdom. The broad strengths and limitations of tobacco industry documents as a data source [ 17, 18], their application to the specific issue of smuggling [ 7], and the distinctive value of, and problems posed by, the Guildford Depository [ 19, 20] have been previously described. Research for this paper was based on an iterative search strategy, commencing with searching the depository's crude file index in 2003 using the keywords China, Chinese, PRC (People's Republic of China), Sino*, Peking/Beijing, Shanghai, Canton/Guangdong, CNTC (China National Tobacco Corporation), and Hong Kong. A total of 210 files were identified and reviewed. This search was then supplemented by similar searches between October 2004 and September 2005 of the BAT Documents Archive (BATDA) Web site containing Guildford and additional BAT-related documents dating to 2003, exploiting the increasing online availability of BAT documents [ 20]. Documents used in this analysis date from 1983–1999.

BATDA also enabled advanced Boolean searches via combined keywords, including well-recognised euphemisms for smuggling defined within BAT documents ([ 21, 22]; available as Text S1 and S2, respectively), such as “transit”, “general trade” ( “GT”), “duty not paid” ( “DNP”), and “free market” [ 7]. A total of 2,594 online documents were reviewed. From both on site and online sources, 882 relevant documents on China and 2,502 on Hong Kong were indexed on a specially designed project database to enable the construction of an historical narrative. Document analysis incorporated several validation techniques within a hermeneutic process [ 23], including corroboration of interpretation between authors and attempted triangulation of findings via trade publications, newspaper articles, academic journals, and other secondary sources.

## Results

### Circumventing Barriers to Market Entry

The Chinese tobacco market remained closed to foreign companies for more than three decades, but in the late 1970s the “open door” policy reintroduced potential access to this market. Confronted with tight import quotas and high tariffs, however, transnational tobacco companies (TTCs) made limited progress in the world's largest market via legal trade [ 24]. Under Chinese law, the State Tobacco Monopoly Administration is the sole agency allowed to trade in tobacco. A special agency under the State Tobacco Monopoly Administration runs all imports and exports of tobacco leaf and cigarettes. No individuals, state, or private enterprises are permitted to engage in this trade. State permits or licenses are needed for all imports, which are subject to tariffs. By the late 1980s, quantities of smuggled cigarettes confiscated rose markedly, although they represented only a fraction of the total contraband, estimated at 5%–10% of China's annual production. Between 1988 and 1992, 26,100 million sticks were seized by customs officials [ 25]. In 1998, Premier Zhu Rongji initiated a national anti-smuggling campaign, resulting in regular seizures of millions of sticks [ 11, 26, 27].

Within this context, industry documents suggest large-scale smuggling of foreign cigarettes into China to circumvent barriers to market access. Official trade figures and other documents cite levels of legal imports, subject to State Tobacco Monopoly Administration quotas and tariffs (180% in 1985) and sold by licensed retailers, duty-free shops, and so-called friendship stores largely frequented by foreign visitors. Comparing official data for selected years, which represent imports by all TTCs, to BAT's sales figures to China reveals dramatic discrepancies ( Table 1). For example, during 1990, official imports from all TTCs totalled 10.5 billion sticks, whereas BAT documents describe company exports to China of over 20.3 billion sticks. The company recognised this trade as “one of the larger profit centres in BAT Industries” [ 28], with 25% of BAT's profits coming “from transit trade to China” [ 29].

**Table 1:**
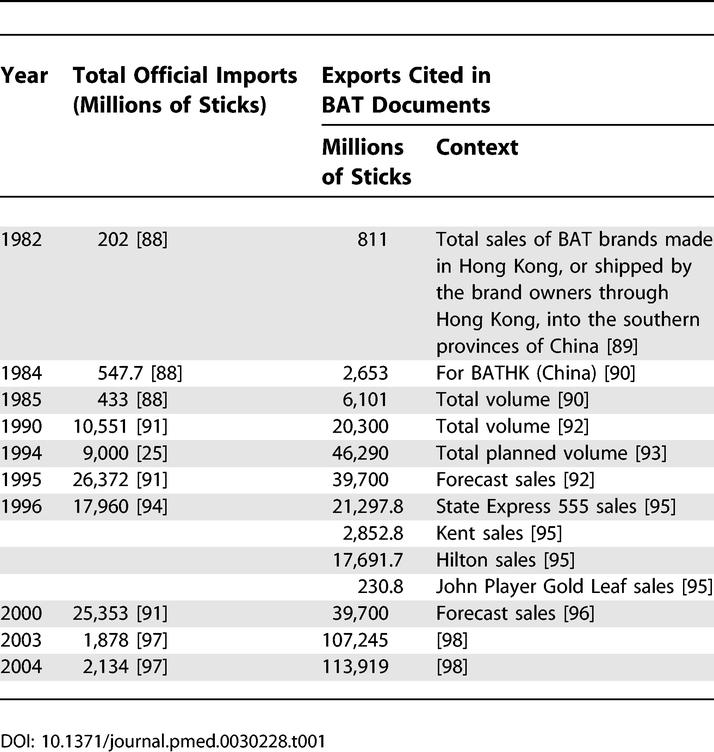
Official Chinese Imports and Cited BAT Exports to China for Selected Years between 1982 and 2000

The difficulty in accounting for such discrepancies is evident in a numbered list of questions and answers prepared by BAT's corporate communications department to prepare staff for answering potential questions about the proposed restructuring of BAT operations in China (discussed below). While direct questions about smuggling were answered with claims that high taxation caused such activity—a commonly recited argument unsupported by independent empirical evidence [ 1, 2, 30]—no response was offered regarding the divergence between Chinese and BAT import figures:

11. Are you aware that cigarettes are smuggled into some Far East markets, particularly China

A. Yes! We are aware cigarettes are smuggled in some parts of the world. This is usually a result of tax differentials, providing an incentive for some to trade illegally in our products. We are opposed to such activities because they have an adverse impact on our legitimate sales…

15. How do you explain the differences in figures quoted by your company (M Broughton presentation) regarding exports to China and officially published figures by the government of PRC?

A. [ 31]

### Restructuring to “exploit opportunities for the sale of BAT Group products to private traders selling into China” [ 32]

Control of the contraband supply chain was essential to optimise BAT sales within different markets, and to avoid unwanted attention from customs and excise officials [ 33]. BAT initiated a restructuring of its China and Hong Kong operations in 1990 “to improve the effectiveness of the Group's penetration of the China market” [ 34]. Problems over supply routes (see Protocol S1 for detailed discussion of supply routes for smuggled cigarettes) were seen to require stronger central management of the export trade:

The reasons for transferring the business from Hong Kong to UK include the increasing need to control centrally the distribution and sourcing of the Group's international brands [ 35].

The impending handover of Hong Kong to Chinese rule in 1997 was a particular concern. BAT Hong Kong (BATHK) was responsible for the Hong Kong and Macau domestic markets, as well as what is described as “combined exports” to China. Sales to China in 1991 accounted for “the largest part of the [BATHK] business…representing approximately 92% of the total” [ 36]. The existing structure of BAT operations in the region was seen as unsatisfactory by David Leach (Project Co-Ordinator, Far East Sales Unit [FESU], BATHK):

There is inadequate segregation of the management of the various markets currently served by BAT HK, marketing activities are inadequately co-ordinated and the structure is insufficiently flexible to respond to changes which may occur in the lead up to 1997 [ 36].

Reorganisation was seen as necessary to facilitate further expansion of the contraband trade across Asia. As Roy Salter (Group Planning Coordinator, BAT Industries) wrote, “Opportunities will emerge for sales to traders dealing in other markets, e.g. Thailand, possibly in conflict with established BATUKE [BAT United Kingdom and Export] and B&W [Brown and Williamson] agents, such as SUTL [Singapura United Tobacco Limited]” [ 37].

In 1990, a report was commissioned from consultancy firm Griffiths Management Limited to develop management and corporate structures that would provide BAT “with a competitive advantage over other international manufacturers” [ 38] in China. The subsequent report envisaged a 3-fold division of responsibility. First, BATHK would be restricted “to manufacturing and selling in the [Hong Kong] domestic market and to manufacturing under contract for other markets as required” [ 39]. Second, BAT China would be established, with responsibility for “direct [legal] imports to China, for managing the relationship with CNTC and for establishing a strong presence for BAT brands within the China market” [ 39].

The proposed restructuring would be completed with the creation of “an offshore company…domiciled in the UK” to handle “selling to trading organisations supplying China and other adjacent markets”. The offshore company was provisionally named BAT Distribution, and later referred to as International Tobacco (Overseas) Limited, and the report was explicit in the description of its proposed remit:

The transit trade for China, Taiwan and elsewhere in Asia should be undertaken by BAT Distribution Limited, incorporated in a tax haven of choice, probably UK. It could be owned by BAT Industries or whatever other company BAT Group prefers.

BAT Distribution should be little more than a brassplate company with very low overhead and the flexibility to establish branch offices wherever the transit traders move [ 38].

The primary objective of this complex model was to provide an operational divide between legal and illegal trade while, critically, ensuring clear oversight and control:

BAT Distribution is set up to enable BATHK to effect a formal separation of its transit from its official trade by transferring the transit trade to this company. BAT Distribution activities remain fully controlled by the BAT China Group (and not any of its subsidiaries) but structurally and formally, BAT Distribution is owned elsewhere in the BAT Group in a tax haven [ 40].

The strategic need for this separation is reaffirmed by BAT executives in discussing procedures for handling orders, invoicing, payment, and supply [ 41]. The need for the contraband trade to be carried out “on an arms-length basis” [ 42], which “removes BAT HK as an intermediary” [ 42], was particularly noted. The company would “seek to identify trading organisations which might have the potential to function in a way similar to that of SUTL [Singapura United Tobacco Limited]” [ 39]. The Griffiths report distinguished this approach from what it termed “the Philip Morris model”, seen as reducing both profitability and control over the transit trade, while noting that the proposed approach would not reduce BAT's ability to “pretend ignorance” of complicity in transit:

It has also been suggested that the Philip Morris model be adopted which places an intermediary between Philip Morris Asia and the transit traders. We have two objections. First the transit trade still originates with Philip Morris Asia and is controlled by that company. That is similar to the present situation with BATHK except for the intermediary. The second objection is that the intermediary adds another level of profit absorption and is harder to control than a dedicated group company. The apparent distancing has little effect since the business is transit trade. All the customers BATHK deals with are legitimate, properly incorporated businesses. Provided this remains the case the BAT Group can pretend ignorance that its cigarettes are being distributed through the transit trade as much and as justifiably as Philip Morris can [ 38].

**Figure 1:**
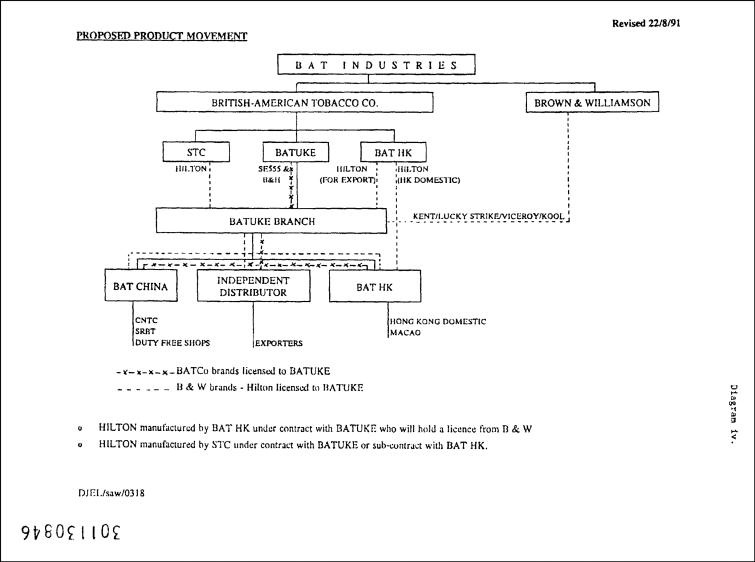
Planned Restructuring of BAT Operations to Separate Supply of “Independent Distributors” to China Source: British American Tobacco Documents Archive. Available: http://bat.library.ucsf.edu/pageview?a=img&tid=fm41a99&total=2. Accessed 19 June 2006.

Figure 1 illustrates the planned restructuring of BAT's China operations, with the contraband trade to be carried out via “exporters” under the responsibility of an “independent distributor”.

This proposed restructuring was submitted to BAT Chairman Sir Patrick Sheehy in March 1991 by former Chairman Barry Bramley, with the reported support of future Chairman Martin Broughton. The paper recommended that BAT Distribution be established with the following responsibilities:

(a) To manage and to exploit opportunities for the sale of BAT Group products to private traders selling into the China, and adjacent general trade markets, in a regulated manner, (effectively replacing the current role of BAT(HK)).

(b) Identification of new trading organisations with the potential to supply China and adjacent markets.

(c) To ensure the efficient distribution within the China markets of duty free BAT products [ 32].

Sheehy and fellow directors approved the reorganisation in April 1991, with BAT Distribution more discretely referred to as “responsible for sales to trading organisations supplying China and other adjacent territories” [ 34].

The planned reorganisation was then reviewed by in-house staff and Price Waterhouse in terms of the complex tax implications for BAT. Senior management sought to identify the optimal location of the three proposed companies to minimise tax liability, noting the “risk that the [UK] Inland Revenue may examine the operation carefully as a result of the overall reduced taxable profits” [ 37].

Given such considerations, a revised restructuring proposal, written by project co-ordinator David Leach and supported by Bramley and BAT Far East Director (now Chief Executive) Paul Adams, was submitted in November 1991 [ 43]. This proposal advanced a simplified model under which “the role of servicing the China market and Hong Kong exports will be taken over by two new organisations, BAT China and a Far East Sales Unit (FESU) attached to BATUKE”. BAT China was to be wholly owned by BAT and have “responsibility for sales of cigarettes to China National Tobacco Corporation, Sino Russian Border Trade (SRBT) and China Duty Free Shops as well as relations between the BAT Group and the Government of PRC”. The FESU, headed by Adams, was to assume the role previously envisaged for BAT Distribution:

FESU, a Division of BATUKE, set up in the UK to co-ordinate the export business currently serviced by BAT HK, and to administer product invoicing and supply from the manufacturers to BAT China, BAT HK and independent distributors. This unit, which will be managed as a profit centre, will be responsible for co-ordinating sales forecasts, arranging contract manufacture and ensuring shipments are made in accordance with schedules [ 43].

This arrangement would enable the group to take advantage of the UK's lower corporate tax rates [ 43], and facilitate central control over China operations. In 1992, total volumes shipped by the FESU to China were 22,889 million sticks, projected to increase to 31,634 million sticks (turnover of US$676.6 million) by 1994 [ 44]. As part of an overall restructuring of BAT operations the FESU was renamed Asia Pacific North in 1993, and then subsequently absorbed under the so-called Project Battalion into the newly formed Asia Pacific Regional Business Unit [ 45].

In seeking to further “disassociate BAT China from the export business” and establish a “structure appropriate for the return of Hong Kong to Chinese control in 1997” [ 46], the contraband trade was to be shifted from Hong Kong to Singapore, from where new supply routes into China were already being explored [ 47]. This entailed BAT's establishment of a “consignment warehouse” in Singapore to enable more flexible and timely delivery to transit agents:

This is because we hope to be able to move the distribution of product currently being sold through Hong Kong exports to the Singapore warehouse which will act as a buffer warehouse in the supply chain. We can minimise costs if we can use ship-to-ship or container-to-container transhipment through the Free Trade Zone in Singapore [ 48].

In July 1993, the warehouse facility opened as “the export supply point for approximately 40% of the combined export sales volume into China”, producing tax savings of £1.5 million (US$998,283) in 1993 ([ 49]; available as Text S3).

### Controlling Prices to “enhance performance”

While official imports were subject to import quotas and tariffs, offering “relative poor corporate profitability” [ 51], BAT stood to earn huge profits by careful pricing of contraband cigarettes. An important consideration was how price could be used to establish brand status within the market. These efforts were apparent for the premium brand State Express 555, which enjoyed “intrinsic exclusivity and prestige” ([ 52]; available as Text S5). The company was keen to build market share, in competition with other “international brands”, but wanted to avoid the threat of “oversupply of SEFK [State Express Filter Kings] to erode price/image” [ 52, 53]. While RJ Reynolds's pricing strategy was closely monitored ([ 54]; available as Text S6), BAT was especially aware of Philip Morris (PM):

As CNTC outlets act as a showcase to Chinese smokers, BAT has ensured that [State Express] 555′s price is positioned at the premium end and above the free market [contraband] price. This deliberate strategy is designed to enhance performance in the free market where the volume is significant. PM adopt a different approach, whereby Marlboro positioned in price both well below 555s [sic] price and below the free market price, has achieved brand leadership in the channel. Despite this, with three key brands BAT will still sell half as much again as PM, with a corporate share estimated at 27% in 1992 (compared to 18.8% for PM) ([ 55]; available as Text S7).

There is evidence that BAT tried to “enhance performance” by controlling the prices of illegal imports ([ 50]; Available as Text S4). BAT thus sought to price its leading brand at a carefully judged premium over those of its competitors, notably PM, intending that State Express 555 should “maintain a 50 cent premium over Marlboro in both the official (CNTC) and Free Market” [ 50], the precise differential varying by channel and region.

The ability to influence pricing of both legal and illegal imports was predicated on control of supply. The challenge was to ensure sufficient supply of BAT brands to compete with domestic and other international brands, without exerting downward pressure on prices. Indeed, the company was aware of the “ability of free market to significantly undercut retail pricing of official duty paid outlets, particularly in the Southern provinces, thereby eroding our potential share performance in official outlets vs free markets” [ 53]. For example, BAT became concerned that the price of Brown and Williamson's premium brand, Lucky Strike, needed to be raised on the “free market”, reportedly “42% below Marlboro” [ 56]. In seeking to control prices, however, the company faced the potential problem of alternative sources of contraband supplying the market:

The CNTC stores are one small source for leakage into the free market….LSF [Lucky Strike Filter] is in the free market in Guangzhou where its price is half that of MARLBORO. The Brand Owner believes that any significant price increase will just suck in transit and that critical mass is needed before any major price repositioning can be embarked on…([ 57]; available as Text S8).

This careful balance between supply and price is described by Kam Ka Ng (Research Manager for BAT China):

We could push as many cigarettes as possible, but to maintain a consistent growth in the long term would require a more strategic approach. We feel that we may be milking the cow, and not feeding it enough to maintain its premium positioning…([ 58]; available as Text S9).

Another complication was the growing presence of counterfeits. By the mid 1990s, there were five sources of foreign cigarette brands in China—cigarettes manufactured locally under licence, legal imports, illegal imports by appointed agents, illegal imports “leaked” from legal or illegal suppliers, and counterfeits. A “significant increase in counterfeit products in China” [ 59] was reported in the late 1990s, and was attributed to the decline in both legal and illegal imports following the Asian financial crisis, which pushed “infringers [counterfeiters] deeper underground” to make “use of unknown transit routes” [ 60]. As Richard Duncan (BATHK) wrote:

Counterfeit has become a major problem in China, causing a loss to BAT in the order of 40 million pounds per annum. Increasingly, there is evidence that China is becoming a source of counterfeit for markets outside China, counterfeiting brands that are not necessarily sold in China [ 61].

It is estimated that around two million cartons of counterfeit cigarettes, both domestic and international brands, were sold in China in 1999, double the amount in 1997. The Chinese government believes that, in many cases, local officials are involved. Raids of factories and workshops during 1998 yielded 3.15 billion counterfeit cigarettes [ 62].

### “It is important that BAT do not get left behind” ([ 63]; Available as Text S10): Smuggling in Pursuit of Global Competitiveness

By the late 1980s, smuggling had become so lucrative that concerns were raised that the pursuit of a joint venture (JV) in China might be counterproductive:

On China, the current view was that the return from exports (including some transit via the USSR) was much higher than from investment. Therefore, a visit by the Chairman [Patrick Sheehy] to Beijing in October (with the ERT [European Round Table of Industrialists]) might actually be counterproductive if he was under pressure to commit to investment [ 64].

The company thus approached JV negotiations with relative caution [ 24]. The strategic question to be answered, according to Paul Adams, was “why are we looking at JV's rather than a continuation of transit...?” [ 65] Estimated sales figures show that “Hong Kong exports” comprised 22% of total sales volume and 27% of total profits for BAT in 1992. These proportions were expected to rise to 29% and 31%, respectively, by 1997 [ 16].

While Hong Kong exports were seen as the “key to the future [of BAT]”, BAT Head of Corporate Planning Graham Burgess recognised the trade's vulnerability given the “danger of serious action by the authorities” [ 16]. In reviewing the BAT company plan for 1993–1997, BAT Group Planning Coordinator Hilary Barton questioned the sustainability of such rapid growth [ 66], while Adams cautioned against excessive reliance on FESU sales:

It is clear that we have relied (and I suspect will continue to rely) on Hong Kong Exports/FESU to meet budget and drive BATCo. growth. I understand and accept the realities of this.

However, it occurs to me that our ability to “make our numbers” through Hong Kong Exports may have caused us to ease the performance pressures off other markets…[ 67].

Progress by competitors seeking JVs in China was also a concern. When PM reached agreement in 1993 for Marlboro to be produced locally, a memo from BAT China's Susan Osborne warned of “serious implications for our GT [general trade] business in China in the medium to longer term” [ 68], while Adams feared “Philip Morris may encourage a G.T. clamp-down” ([ 69]; available as Text S11).

Reliance on an increasingly vulnerable contraband trade in the region shaped the extensive reorganisation of BAT operations in 1993 under Projects Rubicon and Battalion. Given “the increasing ‘globality' of the tobacco business”, the need for structural change was recognised “if BATCo is to fulfil its prime objectives of: (i) growing the value of BAT's brand assets and; (ii) maximising the income stream from each region” [ 70]. The reorganisation was “made necessary, largely because of the spectacular success of the export business in recent years and our expectations that this growth will continue” [ 71]. Combined management was seen as “necessary if we are to remain dynamic and progressive and most importantly, successful in today's competitive business environment” [ 72]. The plan to pursue a twin-track strategy to compete with other suppliers is detailed in a memo entitled “Distribution Initiatives within PRC”:

As long as free market sales remain dominant, alternative routes of distribution of unofficial imports need to be examined, evaluated and, if appropriate, maximised. It is recognised that distribution of our product in China is key to BATCo's long term success and a key objective within BAT China's JV initiative is to achieve widespread distribution networks….

It is important that BAT do not get left behind…BAT would benefit through a wider spread of distribution, particularly in secondary and tertiary cities, ahead of competition…It is envisaged that this initiative would be a short to medium term strategy prior to establishment of any JV operation and naturally any action taken would need to run in parallel with our JV plans…[ 63].

As part of BAT's ultimate goal of becoming “the Premier Tobacco Company in the World” [ 71], the company sought to reach other regions within China, with stated plans to “investigate alternative export routes/customers that will improve direct penetration of UK brands in northern and central provinces” [ 73].

## Discussion

In common with other TTCs, BAT has depicted smuggling as an inevitable consequence of “tax differentials, weak border controls, and import restrictions and bans” [ 74]. In his speech to the BAT Annual General Meeting in 2003, then Chairman Martin Broughton urged governments to enter into partnership with the industry to combat illicit trade:

Governments must consider whether they want the industry to slip into the hands of traffickers and criminals, or to be run by legitimate, tax-paying companies working to manage a risky product responsibly. Many governments recognize that ‘Big Tobacco' is ‘Responsible Tobacco' [ 75].

The documents presented in this paper demonstrate that the separation between “Big Tobacco” and “traffickers and criminals” has been less stark than such posturing would suggest. While public statements by BAT have portrayed smuggling as “inimical to our long-term business interests” [ 76], internal documents illustrate how the company's strategy in China, nonpareil among its future priorities, centred on the supply, oversight, and control of the illicit trade.

The documents demonstrate that contraband has been a hugely profitable and integral part of BAT operations in China over the past two decades. Initially a means of circumventing restricted access to the Chinese market, it became a hugely profitable income stream. Contraband was then used to build market presence, in competition with other international brands, with supply and price carefully managed. By the early 1990s, smuggling into China represented “the largest BATCo market” [ 48], integral to the growth of BAT overall and its aspirations to supersede PM to become the world's leading tobacco company. For the Chinese government, this illicit trade undermined restrictions on imports, represented an enormous loss of tax revenue, and stimulated demand for premium brand cigarettes.

Alongside this lucrative trade, BAT China, deliberately insulated from such operations, sought to build relations with Chinese officials [ 43]. Paul Bingham (Marketing Manager, BAT) noted that BAT China's planned medium-term strategy should address “the ability of BAT to advise the Government generally on tax revenue methods—VAT, excise, direct taxes, oil, alcohol, etc” [ 77]. In 1994, Adams cited the smuggling issue in a discussion with the Chinese ambassador to Britain to gain support for a JV:

Demand for our international brands outstrips the supply via the CNTC Friendship Stores and Duty Free shops. This created a black market. Substantial domestic manufacture by a j.v. would provide China with a share in the profits hitherto taken by smugglers….China had much more to gain with us than without us ([ 78]; available as Text S12).

Yet such lobbying disguises the extent to which BAT has been a critical part of the global smuggling problem, rather than an appropriate partner in its resolution.

Neither BAT nor its senior directors have yet been held accountable, whether via litigation or public inquiry. The United Kingdom has perhaps come closest to pursuing accountability via the concern of the House of Commons Health Select Committee that led to a lengthy investigation by the Department of Trade and Industry. The eventual abandonment of this closed investigation in 2004, and the decision, without publication of its findings, not to pursue a criminal investigation [ 79], has come amid reports of undue political influence exercised on BAT's behalf at the highest levels of government [ 80]. This, alongside the continued accumulation of documented evidence of complicity, raises questions that the Health Select Committee might usefully revisit.

Given the inherently transnational nature of smuggling, collective efforts across countries offer the most promising avenue for developing an effective policy response. The FCTC is particularly significant in this respect, particularly given China's ratification of the convention on 11 October 2005. The first international public health treaty negotiated by the World Health Organization, the FCTC both offers an institutional basis to support the broad development of tobacco control and incorporates a clear focus on tobacco smuggling as a core issue in health policy. Indeed, smuggling received a unique degree of attention via the International Conference on Illicit Tobacco Trade [ 81], reflected in the comparatively detailed coverage afforded the illicit trade via Article 15 [ 82]. Notwithstanding such progress, however, there is a clear need for the broad obligations of the FCTC to be augmented with more specific measures via the negotiation of a dedicated protocol on the illicit trade in tobacco [ 83]. Such a protocol should incorporate, among other things, anti-money-laundering provisions, a mandatory tracking system of markings and codes, records of shipments, and a strict liability and compensation system [ 84, 85]. The basis for such a compensation system might be provided by the conditions accepted by Philip Morris International under its 2004 agreement with the European Union [ 86], a system of binding obligations that contrasts with the weaker voluntary approach of “memoranda of understanding” advocated by BAT [ 87].

## Supporting Information

Alternative Language Abstract S1 Chinese Translation of the Abstract (54 KB PDF)

Text S1 Reference [ 21]
(443 KB PDF)

Text S2 Reference [ 22]
(3.3 MB PDF)

Text S3 Reference [ 49]
(341 KB PDF)

Text S4 Reference [ 50]
(450 KB PDF)

Text S5 Reference [ 52]
(1 MB PDF)

Text S6 Reference [ 54]
(1.9 MB PDF)

Text S7 Reference [ 55]
(2.8 MB PDF)

Text S8 Reference [ 57]
(2.3 MB PDF)

Text S9 Reference [ 58]
(1.1 MB PDF)

Text S10 Reference [ 63]
(1.2 MB PDF)

Text S11 Reference [ 69]
(812 KB PDF)

Text S12 Reference [ 78]
(756 KB PDF)

Protocol S1 Three Supply Routes for Smuggling Cigarettes into China (739 KB DOC)

## Abbreviations

BAT: British American Tobacco
BATDA: British American Tobacco Documents Archive
BATHK: British American Tobacco Hong Kong
BATUKE: British American Tobacco United Kingdom and Export
CNTC: China National Tobacco Corporation
FCTC: Framework Convention on Tobacco Control
FESU: Far East Sales Unit
JV: joint venture
PM: Philip Morris
PRC: People's Republic of China
TTC: transnational tobacco company
