# Correction: “Key to the Future”: British American Tobacco and Cigarette Smuggling in China

**DOI:** 10.1371/journal.pmed.0030390

**Published:** 2006-09-05

**Authors:** Kelley Lee, Jeff Collin

In *PLoS Medicine*, volume 3, issue 7: DOI: 10.1371/journal.pmed.0030228

In Table 1, column 3, last row, the value 113,919 is incorrect; it should be 117,964.

