# Supplementary material for: “Key to the Future”: British American Tobacco and Cigarette Smuggling in China
Source: PLoS Med. 2006 Jul 18;3(7):e228. doi: 10.1371/journal.pmed.0030228 (PMC1502159; doi:10.1371/journal.pmed.0030228)
Supplement: Supplementary file 1 [file pmed.0030228.sd001.pdf]

B.A.T (U.K. and Export) Limited

Export House Woking Surrey GU21 1YB England

Telephone 0483 757555
Telex 859101 BATUKE G
Fax 0483 756100
Telegraphic Address EXPORT WOKING

25th August 1989

MARK SYTA
Frank Small and Associates
Taipei,Taiwan
Fax: 02 5816574

010586

FR A.GEORGE-PERUTZ
MARKETING INTELLIGENCE

RE:TRANSIT STUDY

Many thanks for your fax. Will contact you early next week regarding approval of discussion guide, recruitment questionnaire etc. Would it be possible to delay the start of the Thailand fieldwork for one week and commence on the 12th Sept and for the Taiwan study to begin on the 16th Sept. This allows us time to deliver the stimulus materials and arrange for viewing of the initial groups in both countries. Would also be grateful for the simultaneous translation offered for both countries.

With regard to the definition of transit it is essentially the illegal import of brands from Hong kong, Singapore, Japan etc. upon which no duty has been paid. To my understanding this is most prevalent in the South of Taiwan and I would suggest that the groups are conducted in that region. For better clarification of volumes and incidence call Trevor Ivey on your return.

On the 555 study I appreciate that the locating of 555

302000021

BATCo document for Lon Sch Hyg & Trop Med 26 July 2002
