# Supplementary material for: “Key to the Future”: British American Tobacco and Cigarette Smuggling in China
Source: PLoS Med. 2006 Jul 18;3(7):e228. doi: 10.1371/journal.pmed.0030228 (PMC1502159; doi:10.1371/journal.pmed.0030228)
Supplement: Supplementary file 2 [file pmed.0030228.sd002.pdf]

BRITISH-AMERICAN TOBACCO COMPANY LIMITED

TO: P N Adams

FROM: P C O'Keeffe

DATE: 15 June 1994

SUBJECT: Export Channel Management

I attach a draft of a document being developed for eventual presentation to each Regional Director by their respective Regional Export Manager, trying to define a global approach to Export Channel Management.

I would be grateful for your views as my line boss and the Regional Director as to whether:-

1. Overall is the document coherent?

2. Are the Strategic Principles to which endorsement is being sought clear?

3. Having read it, do you get a feeling that the people managing exports know what they are doing in a strategic sense?

This latter point is key as all the Regional Export managers wish to kill off the "BATUKE Cowboy" image.

Best Regards.

Patrick O'Keeffe

Attch

PCOK/29/fb

MILLBANK KNOWLE GREEN STAINES MIDDLESEX TW18 1DY

503932317

BATCo document for Lon Sch Hyg & Trop Med 14 February 2002

Draft-13/6/94

EXPORT CHANNEL MANAGEMENT

1. Objective

The objective of this paper is:

1.To define a Mission Statement for the Export Channels.
2.To define the sub-channels in the business.
2.To analyse the current situation.(SWOT Analysis)
3.To propose a strategic focus.
4.To seek endorsement from the BATCO board to the strategic principles

2. Mission

To build BATCO's share of the global export business, so as to contribute to the achievement of BATCO's overall volume and profit goals. This will be achieved through the management by BATCo of Exports as a single channel focusing on developing strategic brands in priority markets, but also aggressively exploiting tactical brands where appropriate in support of strategic brands.

3. Definitions

Exports:
In the context of BATCO, Exports are sales of company brands manufactured in one country and sold in another.
In most instances this means sales invoiced through BATUKE, and from 1/1/95 will include BWIT and Souza Cruz brands in those countries for which BATCO is responsible.BATCF brands are currently invoiced through BATUKE for the relevant markets.See Appendix A for definitions on Domestic Markets, GT and Duty Free - the sub-channels of Exports.

4. Scope/Current Situation

It is proposed that the core focus of the Export business be on the development of BAT Industries strategic international brands, it is these with which we will build value share and are also most at risk of "leakage" from one market to another. Therefore while the importance of brands like Hilton and Belmont are recognised for their regional importance, the scope outlined below is principally focused on strategic trademarks.

Brand	1993 Global Vol. Bns	1993 Export Vol.	%
555	22.5	20.6	91.5
B&H	15.3	5.0	32.9
JPGL	11.2	2.7	24.1
Kent	17.7	3.8	21.2
L.Strike	14.8	0.2	1.2
Barclay	4.6	0.0	0.1
Hilton	17.0	2.9	17.2
Belmont	32.2	1.4	4.3

503932318

BATCo document for Lon Sch Hyg & Trop Med 14 February 2002

See Appendix B for SWOT Analysis of Exports and share of BATUKE Exports by RBU.

5. **Priorities**

Each RBU has defined it's priorities in terms of Markets within the respective region and also the Brand Portfolio with which to attack these priorities See appendix C for the global brand priorities split between strategic and tactical brands.

6. **Strategic Principles**

To strategically manage all export routes into each end market so as to increase BATCo's strategic brand's share of total market.

The goal for the strategic brands in each end market is to achieve the retail price positioning required by the brand owners irrespective of the sub-channel of supply. In the short term therefore the volume is restrained by the pricing objective, and in general each market will have exclusivity of supply arrangements via one distributor.

Significant volume gains in the short term will normally be achieved with tactical brands, where volume rather than a specific price point will be the objective;to gain market share, to facilitate distribution of the strategic brands, and to provide the trade with cash so as to be able to carry the stock cost of the strategic brands

To minimise the threat of re-export of strategic brands, but to take calculated risks within overall strategy when focusing on priority markets.

Well established procedures exist to ensure strategic brands are not re-exported. To seek to eliminate the risk altogether would be to deny growth opportunities, particularly in new markets. A more flexible attitude to risk will be taken in those markets defined as top priority in each RBU, still applying the normal control procedures as appropriate.

To adopt an aggressive approach with tactical brand sourcing, price and response time.

The role of tactical brands is to facilitate the growth of strategic trademarks, their volumes alone are not measurements of success. The goal is to use a limited number of marks across all RBU's to fulfill the above role, seeking to identify lowest cost sources of manufacture, not necessarily in the same RBU as the end market.

To identify key customers in each sub-channel and develop long term business building strategies with them.

The customers involved in the Export business are some of the biggest in the Group. The intention is to identify by sub-channel the 20% of customers accounting for 80% of the business,and ensure that there are clear plans in place to have appropriate levels

503932319

BATCo document for Lon Sch Hyg & Trop Med 14 February 2002

of BATCo management contacting them in line with the principles of Trade Marketing. Some customers are involved principally in GT business, but should be managed as professionally as a "Sainsbury's"

To clarify the role of BATCo Regional and Area Management as Distributor Managers for the domestic export and GT markets, and benchmark supplier to the DutyFree Channel.

This implies:

Identifying and monitoring the profitability by channel, region, market and customer.

Implementing performance related distributor/customer contracts.

Setting goals for improving BATCo profitability through improving efficiency in the Trading Chain, so that the cost savings achieved can be shared between the customers and BATCo.

Identifying the training needs of BATCo management, and implementing appropriate programmes, e.g.

Negotiation Skills
National Account Skills
Competitive Edge Skills
Presentational Skills

Distribution
Warehousing
Freight
Finance

To adopt best practises across RBU's
Through the mechanism of the Export Meeting (the monthly meeting of all REM's) to ensure that successful learning is transferred across RBU's

7. __Pricing__

Strategic Brands will be positioned in line with IBG guidelines in end markets.
Control will be through SEM sign off on Terms of Trade changes, and monthly monitoring of all customers selling strategic brands below a benchmark Nett Nett Price.

Tactical brands will be sourced and priced flexibly so as to achieve the strategic goals.

End market pricing will be continuously monitored, as will Trade Terms and Conditions.

We will seek to implement CIF terms as standard.
This will reduce likelihood of leakage.

503952320

BATCo document for Lon Sch Hyg & Trop Med 14 February 2002

8. Controls and Security

A review will be undertaken by the Export Meeting to ensure adequate security is achieved. The current situation is unacceptable in terms of too widespread availability of commercially sensitive information,and specific proposals to correct the situation will be presented to the Export Meeting in Q3 1994.

9. Recommended Action

1.BATCo Board to endorse the Strategic principles outlined above.

2.BATCo Board to hold responsible REM's for delivery of the strategic principles,with the Senior Regional Export Manager responsible for overall co-ordination.

3.Plans to be prepared by RBU in line with Priority Market/Brand definitions

4.Quarterly progress reports to be presented to the Export Meeting

503932321

BATCo document for Lon Sch Hyg & Trop Med 14 February 2002

Appendix A

Definitions

Domestic Markets are those where exports are made to well established customers in countries where the product is sold duty paid, usually with country/territory specific pack markings, for consumption primarily within that country.

GT refers to exports made for onward sale to another market other than the market to where product was shipped, and where the packaging would normally be non market specific. Such products would often have substitute coding to identify the customer and therefore the intended end market.

Duty Free refers to exports to the operators or concessionaires of facilities enabling them to sell the product without the inclusion of duty/excise in the price. In most cases there are no specifc markings required. In some instances DF product penetrates into the local domestic market, and in this context DF business can be regarded as GT.

503932322

BATCo document for Lon Sch Hyg & Trop Med 14 February 2002

Appendix B (1)

SWOT of Current Export Business.

Strengths

- Highly profitable.
- Tax efficient through BATUKE.
- High volume, and growing.
- Significant levels of Marketing Support.
- GT business tightly controlled.
- Portfolio of UKIBs and USIBs.

Weaknesses

- Ill defined overall strategy for all channels in combination.
- Uncoordinated approach to opportunity markets across regions.
- Fragmented portfolio for opportunity markets.
- Aversion to risk.
- Business not evaluated by consumer value share in end market.
- Poor understanding of competitors strategies, plans and activities.

Opportunities

- Untapped markets present growth opportunities.
- US IB's in portfolio.
- Utilisation of GT to build IB business in priority markets where applicable.
- Mix strategic and tactical brands to build business.
- Utilise OpCos for sourcing low cost tactical brands.
- Team approach to problem solving.

Threats

- Volatile nature of GT business.
- Sunday Times factor.
- Destructive effect of re-export and parallel trade.
- Counterfeit.
- Competition strategically managing exports better than BAT Co.

503932323

BATCo document for Lon Sch Hyg & Trop Med 14 February 2002

Appendix B (2)

EXPORT BUSINESS - Baliuko invoiced shipments

RBU		1992 Mns	1993 Mns	Jan - Mar YTD 1994 Mns
Africa	Dom	1,189	1,511	300
	D F	102	86	25
	GT	1,226	1,137	197
		2,517	2,734	522
ME	Com	3,916	4,060	1,382
	D F	155	150	35
	GT	8	12	0
		4,079	4,222	1,417
AP	Dom	4,209	4,841	1,981
	D F	3,018	3,820	1,691
	GT	20,993	22,389	7,026
		28,220	31,050	10,698
EU	Dom	1,968	1,639	236
	D F	1,806	2,001	418
	GT	0	23	4
		3,774	3,663	658
LA	Dom	547	469	122
	D F	157	120	11
	GT	8	1,401	1,018
		712	1,990	1,151
Total	Dom	11,829	12,520	4,021
	D.F	5,238	6,177	2,180
	GT	22,235	24,962	8,245
		39,302	43,659	14,446

Regional Summary 93				
Africa	2,734	6 26%	522	3 61%
ME & ISC	4,222	9 67%	1,417	9.81%
AP	31,050	71.12%	10,698	74.06%
EU	3,663	8 39%	658	4 55%
LA	1,990	4 56%	1,151	7 97%
	43,659		14,446	

Dom Summary 93				
Africa	1,511	12 07%	300	7.46%
ME & ISC	4,060	32 43%	1,382	34 37%
AP	4,841	38 67%	1,981	49.27%
EU	1,639	13.09%	236	5.87%
LA	469	3.75%	122	3 03%
	12,520	28.68	4,021	27.83

D.F Summary 93				
Africa	86	1.39%	25	1.15%
ME & ISC	150	2.43%	35	1.61%
AP	3,820	61.84%	1,691	77.57%
EU	2,001	32 39%	418	19.17%
LA	120	1.94%	11	0.50%
	6,177	14.15	2,180	15.09

GT Summary 93				
Africa	1,137	4 55%	197	2 39%
ME & ISC	12	0 05%	0	0 00%
AP	22,389	89 69%	7,026	85.22%
EU	23	0 09%	4	0.05%
LA	1,401	5.61%	1,018	12.35%
	24,962	57.17	8,245	57.07

GT Breakdown		
HK	11,130	44 59%
Singapore	8,237	33 00%
FE Others	3,022	12 11%
Aruba	1,401	5 61%
Niger	989	3 96%
Others	183	0.73%
	24,962	

Note: These are unadjusted figures i.e HK & China DF are categorised as DF, Djibouti & Guinea are classified as Dom. The effect of changing these to GT is to increase the proportion of GT by 4.65 bn, or from 57 % to 63 % of the total business.

14-Jun-94

503952524

BATCo document for Lon Sch Hyg & Trop Med 14 February 2002

Appendix C

Export Brand priorities - Global

	High	Medium	Low
**Strategic**	*State Express*<br>*B & H*<br>*JPGL*<br>*Kent*<br>*Lucky Strike*<br>*Barclay*	*JPS*<br>*Capri*<br>*Kool*<br>*Silk Cut*	
**Tactical**	*Hilton*<br>*Viceroy*<br>*Embassy*<br>*Pall Mall*<br>*Belmont*<br>*Gladstone*<br>*Derby*<br>*WGF*<br>*Premium*	*Capstan*<br>*Finesse*<br>*Advance*<br>*Ardath*<br>*Casino*<br>*Richland*	*L & B*<br>*AC No.1*<br>*Senior Service*<br>*du Maurier*<br>*Players*<br>*Others*

503932325

BATCo document for Lon Sch Hyg & Trop Med 14 February 2002

Export Brand priorities - Asia Pacific

	High	Medium	Low
**Strategic**	*State Express*<br>*B & H*<br>*JPGL*<br>*Kent*<br>*Lucky Strike*<br>*Barclay*	*JPS*<br>*Capri*<br>*Kool*<br>*Silk Cut*	
**Tactical**	*Hilton*<br>*Viceroy*<br>*Embassy*<br>*Pall Mall*	*Capstan*<br>*Finesse*<br>*Ruby Queen*	*AC No.1*

503932326

BATCo document for Lon Sch Hyg & Trop Med 14 February 2002
