# Supplementary material for: “Key to the Future”: British American Tobacco and Cigarette Smuggling in China
Source: PLoS Med. 2006 Jul 18;3(7):e228. doi: 10.1371/journal.pmed.0030228 (PMC1502159; doi:10.1371/journal.pmed.0030228)
Supplement: Supplementary file 3 [file pmed.0030228.sd003.pdf]

- Review of BATCo's Regional Manufacturing Strategy in Asia Pacific which resulted in the change from double to single shift operation in BAT Hong Kong at end December 1993. The restructure resulted in the loss of 120 jobs at a cost of HK$10mn. The annualised payroll savings from this manufacturing downsizing are HK$18 mn.

- With BATCo being designated the responsible Company for the Indochina and Thailand markets, two from a total of seven Managers employed by B&W for these markets were recruited by BATCo to reflect the additional volumes from the B&W portfolio: a net saving of 5 Managers to the BAT Industries Group. This has been absorbed into the existing RBU management without any additional resources at the centre.

- Additional initiatives in 1993 have been the establishment of a new warehouse facility in Singapore, opened in July 1993 which is now the export supply point for approximately 40% of the combined export sales volume into China. Tax savings as a result of this move amount to £1.5 mn in 1993. Further distribution points are being explored in order to offer increased flexibility of supply to our export customers.

- Financial controls within the export offices of Singapore and Taiwan have been strengthened with plans to appoint Finance Managers in these operations.

- The appointment of a CEO for Asia Pacific North for the Hong Kong, China and Taiwan markets has been introduced and this has resulted in the planned integration of the BAT Hong Kong and BAT China Marketing and Duty Free departments in February 1994.

503952873

BATCO CONFIDENTIAL - CATEGORY I: MINNESOTA TOBACCO LITIGATION.

BATCo document for Lon Sch Hyg & Trop Med 12 February 2002
