# Supplementary material for: “Key to the Future”: British American Tobacco and Cigarette Smuggling in China
Source: PLoS Med. 2006 Jul 18;3(7):e228. doi: 10.1371/journal.pmed.0030228 (PMC1502159; doi:10.1371/journal.pmed.0030228)
Supplement: Supplementary file 4 [file pmed.0030228.sd004.pdf]

Pricing Strategy

1) SE555

a) General

The high price relative to other mainstream brands is a major factor in the success of 555, re-inforcing the strong status and prestige values and high product quality.

555 must therefore maintain its intrinsic premium pricing position over other leading mainstream competition. If this premium is lost, these values will be eroded.

b) Retail pricing - Free Market

- SEFK

To ensure that the essential retail pricing premium of non-HKDNP marked stock of at least Rmb 0.40 - Rmb 0.50 per pack is maintained over major mainstream competition in the south i.e. Marlboro KS SC. In central and northern areas, this differential would expand to 1 Rmb per 20.

HKDNP marked stock should always retain a Rmb 1.00 - 1.20 premium over Marlboro KS SC in southern markets, although generally retaining parity with non-HKDNP product in the central and northern markets, because of differing perceptions of such product in these areas.

SE555's pricing in relation to short supplied local high priced brands such as Yun Yan, Hong Ta Shan, Ashima, is also key to brand growth. SEFK's target pricing should be to maintain price band parity with these local high priced brands.

- SE International

It is planned that a new SE555 International variant will be introduced through CNTC and HK exports in Q1 1993. Retail pricing should be targeted at achieving a 20's packet price of Rmb 10.00 - 11.00 in the south.

It is accepted that large volume will not be realized with this variant in the short to medium term, however the exercise will serve to further emphasize SEFK's prestige, and may eventually help to raise retail pricing levels and consequently further re-inforce status and quality values of SEFK.

301591553

BATCo document for Mayo Clinic 17 June 2002
