# Supplementary material for: “Key to the Future”: British American Tobacco and Cigarette Smuggling in China
Source: PLoS Med. 2006 Jul 18;3(7):e228. doi: 10.1371/journal.pmed.0030228 (PMC1502159; doi:10.1371/journal.pmed.0030228)
Supplement: Supplementary file 5 [file pmed.0030228.sd005.pdf]

III. UK BRAND STRATEGIES - CHINA

A. OVERALL MARKETING OBJECTIVES

1) Over the plan period to further grow SE555's dominant share from 13.6% in the imported segment in the duty paid and duty free markets. SE555's position can be further improved with additional variants under research, to achieve a target family share of 17% in 1996.

2) To maintain and emphasize SE555's intrinsic exclusivity and prestige.

3) To achieve the following levels of unaided advertising awareness (total mentions) for SE555 (total) mentions by end 1992:

Beijing	-	25%	Current (10%)
Shanghai	-	20%	Current (3%)
Guangzhou	-	70%	Current (18%)

4) To further augment UK brand share growth, to gain a minimum incremental 2.0% share of imports at the end of the plan period utilizing other viable UK trademarks either currently available or to be researched. Priority attention should be given to the mid price segment (25%) where the group portfolio of brands need strengthening. However, such entries must not encourage cannibalization of SE555.

5) Achieve a broad degree of UK brand portfolio consistency with neighbouring markets in Taiwan, Hong Kong, and Indo China markets.

6) To use the Hong Kong domestic market as a showcase for 555 to underline the international appeal.

7) To maximize all appropriate A.T.L. and B.T.L opportunities for existing and new brands, cost effectively, to brand guidelines.

B. OVERALL MARKETING STRATEGIES

1) Maintain SE555's prestigious position and premium pricing differential.

2) Consider and research JPS White KS Box as mid price entry that has the ability to gain totally incremental volume.

3) Place the B&H trademark in the full price segment for controlled exploitation.

301591545

BATCo document for Mayo Clinic 17 June 2002

4) To implement visibility and trial programmes for SE555, parent and line extensions, and any other viable new brands, in the Hong Kong domestic market,in liaison with BATHK.

5) To identify suitable reputable advertising agency resource(s) that are well established in China, that provide a national service, are of international reputation, and are acceptable to IBM unit.

301591546

BATCo document for Mayo Clinic 17 June 2002

SE555 CHINA

I. S W O T ANALYSIS -

1. STRENGTHS

- SEFK Exclusivity - function of pricing/heritage/perceived status
- SEFK volume/share and resultant profitability
- SEFK distribution/offtake in Southern Provinces (est 50-60% of total volume)
- BATHK relationships with CNTC
- Branding impact

2. WEAKNESSES

- Poor market knowledge/data gathering
- Historical marketing resource - resultant poor advertising awareness levels
- Manpower resource in PRC - quality - sales and merchandising functions
- Historical support given to SEFK
- Comparatively poor performance of SEFK in North & Central
- 'Operator' Policy
- Use of SEFK as volume 'Tool'
- SE555 Brand management - Consistency
- Brand family (variants)

3. OPPORTUNITIES

- Revitalize SEFK advertising and awareness
- Focussed marketing effort - Planning
- Effective use of reputable agency resource
- Improve volume/share/profit performance in CNTC - develop contingency plans
- SEFK - Pricing vs Volume
- SE Int'l - emphasize prestige
- SE100's - incremental volume - develop trial & availability -> new design
- SE555 Lights - HK dom -> China
- Create new/improved routes into China (North direct)
- Increased restrictions - benefit to 'core' brands.

4. THREATS

- PRC enforced moves to halt GT
- Oversupply of SEFK to erode price/image
- Parallels e.g. Cyprus, Singapore
- Counterfeit product
- Weakening SEFK performance in HKG - affect on Southern China
- Increased advertising restrictions eg. TV sponsorship
- Erosion of consumer perceptions

301591547

BATCo document for Mayo Clinic 17 June 2002
