# Supplementary material for: “Key to the Future”: British American Tobacco and Cigarette Smuggling in China
Source: PLoS Med. 2006 Jul 18;3(7):e228. doi: 10.1371/journal.pmed.0030228 (PMC1502159; doi:10.1371/journal.pmed.0030228)
Supplement: Supplementary file 6 [file pmed.0030228.sd006.pdf]

Seen D.
File - China
Marketing

B.A.T. CHINA LIMITED
31st Floor, One Pacific Place, 88 Queensway, Hong Kong
Telephone : 514 2188 · Fax : (852) 877 6428

FACSIMILE TRANSMITTAL

OUR FAX NO. : (852) 8776428	YOUR FAX NO. : 002-44-784-448390
TO : BATCO	ATTENTION : P. N. Adams
FROM : Mr. N. Lovett	C.C. : I. G. Hacking (#784-448395)<br>P. C. O'Keeffe (#784-448395)
DATE : 7 June 1993	NO. OF PAGES : 4<br>(including covering page)

PHILIP MORRIS PRICING STRATEGY

With reference to your note of 11th May to Hoe Seong on the above, pages 2 - 4 detail BAT China's response. This response addresses possible PMI scenaria only.

Given RJR's reported hunt for cash, as evidenced in Hongkong, it is likely that pricing of their key brands would be held, and a price reduction response would be unlikely, regardless BAT's eventual action. However we believe that RJR's stance would be unlikely to adversely impact key BAT brands.

Regards,

NAL

Neil Lovett

Encl.

NAL/rs

500014814

BATCO CONFIDENTIAL - CATEGORY I: MINNESOTA TOBACCO LITIGATION.

BATCo document for Lon Sch Hyg & Trop Med 12 February 2002

Contingency Strategies re: Marlboro Pricing in China:

Background:
BAT China have been asked to propose contingency strategies in the event that Philip Morris should institute significant pricing initiatives for their premium brand Marlboro.

For SE 555, pricing is a fundamental part of the product mix and impacts, in China, upon the image perceptions of the brand. As such any decision to reduce the price has to be taken in the light of additional factors apart from purely competitive initiatives. For 555, the stated pricing strategy and objective has been to maintain a 50 cent premium over Marlboro in both the official (CNTC) and Free Market. However over the past few months this pricing objective has not been met due to a) an undersupply against demand of Marlboro in the export/free market and b) the devaluation of the RMB against HK$. In official markets 555 has still managed to maintain a pricing advantage over Marlboro KS SC and in the duty free area the pricing is currently at parity.

For Kent a key objectives in the near term is to attempt to increase the free market price to a level nearer to that of the official market price through a managed reduction of volumes supplied through the export channel. Through April and early May some improvement in free market pricing was noted.

Lucky Strike has been withdrawn from the export market. Some stocks are still present in the free market but the focus of efforts has been on maintaining official price at current relativity versus Marlboro. i.e. One price category below Marlboro.

Hilton and Viceroy currently both occupy the low price segment of the International brand market. Hilton particularly is under priced in the free market versus the official market and it's overall positioning is currently under review.

Recent pricing points for each of the key brand, in the key cities, is set out below:

Free Market Pricing (RMB)Brand	Guangzhou	Shanghai	Beijing
Marlboro	8.1	9.0	9.5
555	7.8	9.0	9.5
Kent	6.8	7.5	7.0
Hilton	4.0	5.0	5.5
Lucky Strike	4.2	5.0	5.5
Viceroy	4.2	5.0	4.5

Official Market Pricing (FEC)Brand	Guangzhou	Shanghai	Beijing
Marlboro	5.7	6.7	7.5
555	6.0	7.6	7.5
Kent	5.3	6.7	7.2
Hilton	3.5	4.3	4.5
Lucky Strike		5.2	6.2
Viceroy			

*NB FEC premiums applied to achieve Rmb as folows: Beijing - 30%, Shanghai - 50%, Guangzhou - 67%

500014815

BATCO CONFIDENTIAL - CATEGORY I: MINNESOTA TOBACCO LITIGATION.

BATCo document for Lon Sch Hyg & Trop Med 12 February 2002

The key ways in which Marlboro can achieve price reductions are as follows:

a) CNTC retail pricing reflects the the desired benchmark price positioning for all brands Through reducing the price to CNTC/CNDF sufficiently to engineer a reduction in their retail price (assuming that CNTC do not merely take the increased margin) to help compete with free markets. This however is dependant upon their ability to re negotiate their contract with CNTC which could prove extremely difficult or to offer significant rebates and gratis in order to achieve the same objective within their current contractual commitments; this would be likely to set undesirable precedents. BAT will need to closely monitor.

Philip Morris would be more likely to opt to renegotiate prices down in the 1994 contract negotintions in order to achieve pricing reductions. The 1994 contract negotiations will need to be carefully conducted with agreed industry stances and constant dialogue with CNTC in order to ensure that we are offering competitive terms to achieve our target price relativity.

b) To reduce the price to exporters, which, although likely to increase margins in the short term, could, through the probable increased supply into the domestic market, result in an oversupply of the brand and a decrease in the free market price

If Marlboro pricing is reduced, with the objective of reducing free market retail prices, and enhancing margins it is essential that our own export prices be addressed quickly in order to ensure that our in market sales volumes and trade margins are protected.

500014816

BATCO CONFIDENTIAL - CATEGORY I: MINNESOTA TOBACCO LITIGATION.

BATCo document for Lon Sch Hyg & Trop Med 12 February 2002

Proposed Actions:

BAT China Response/Options	PM Action<br>Reduce the price of Marlboro in Official Markets
555	1. Maintain target price premium against Marlboro following down as necessary.
Kent	1. Maintain price until parity pricing is achieved then follow Marlboro down maintaining price parity
Lucky Strike	1. Maintain price until price differential of 10 - 15% is achieved. The current differential is between 20 - 25%. Once the desired level of pricing is achieved follow Marlboro down maintaining the planned differential
Hilton	1. Recent experience has indicated that the price / volume relationship between Hilton and Marlboro is closely linked. Hilton pricing should be continually reviewed -v- Marlboro. Reduce price to maintain differential but ensure that price does not fall below that of the competitive local brand set i.e. c4.0 RMB
Viceroy	1. Reduce price to maintain differential
BAT China Response/Option	PM Action<br>Reduce price of Marlboro in Free Market
555	1. Maintain price, initially allowing premium to be extended. Assess impact on consumer offtake within 2/3 weeks, and take corrective action to redress if necessary.
Kent	1. Should price of Marlboro remain above that of Kent maintain Kent pricing and assess impact on sales through sales force monitoring
	2. Should Marlboro reduce price to parity with Kent, maintain current price and assess impact upon sales. Should Kent sales drop significantly decrease Kent to 90% of Marlboro price
	3. Should Marlboro reduce price to below that of Kent, reduce price of Kent initially to parity, assess sales impact, and, if necessary, reduce to 90% of Marlboro pricing
Hilton	1. Hilton and Marlboro sales are currently price/volume linked. Assess effect of Marlboro price reduction on Hilton sales and be prepared to reduce price but maintain a price premium over the relevant free market local brand set. Retail price not to fall below 4.0 RMB
Viceroy	1. Reduce price to maintain relativity with own/competitive brands.

Price Elasticity:

We are currently instituting a BPTO model (scheduled for July 1993) to assess the elasticity of major brands in the market. It may well be that at current prices Marlboro already has little elasticity, which is beginning to show through from our own audits, but there is a need to be able to assess where, following a price decrease, any additional volumes for Marlboro will come from and which are the vulnerable brands e.g. Kent and or Hilton, 555 etc. Similarly an elasticity study on 555 will assess the impact of reduced pricing of Marlboro on our own franchise and could well indicate that, regardless of pricing strategy the current absolute level of pricing for 555 is sufficiently robust to maintain current volumes even allowing for a wider price differential between the two brands.

500014817

BATCO CONFIDENTIAL - CATEGORY I: MINNESOTA TOBACCO LITIGATION.

BATCo document for Lon Sch Hyg & Trop Med 12 February 2002
