# Supplementary material for: “Key to the Future”: British American Tobacco and Cigarette Smuggling in China
Source: PLoS Med. 2006 Jul 18;3(7):e228. doi: 10.1371/journal.pmed.0030228 (PMC1502159; doi:10.1371/journal.pmed.0030228)
Supplement: Supplementary file 7 [file pmed.0030228.sd007.pdf]

File - China Marketing   Mr. P.N. Adams

BRIEFING NOTE   P.A.

PHILIP MORRIS IN CHINA   28 AUG 1992

COMPANY SHARE

• BAT, the leading foreign cigarette Company in China is 3 times the size of PM, No. 2 in the market. In 1992, BAT's dominant share, estimated at 54.3% of total cigarette imports, relates to that of PM of 16.6%.

• In 1991 and, more particularly, 1992 BAT has significantly outperformed PM in share growth. In 1991, BAT's share grew 3.9 share points (to 49.0% of share) compared to PM's 2.4 share points (to 17.2%) in the imported segment. On the back of a strong performance by 555, BAT's share growth in 1992 is expected to further outstrip PM's performance, increasing by 5.3 share points to 54.3%. In 1992, due mainly to a softness in Marlboro, PM's share is expected to decline by 0.6 share points to 16.6%.

BRAND PERFORMANCE

• While a portfolio of three major brands (555, KENT, and HILTON) contribute to BAT's dominance in China, Philip Morris' business is heavily dependent on Marlboro, which brand accounts for 89% of total PM volume.

• As a result of continuing strong growth in 1992, 555 has now become the No.2 imported brand with a share of imports of 14.9% for the year to date to July, compared to Marlboro which will have eased to a share of 13.5% for the same period. Hilton remains the market leader in the imported segment growing to an estimated 26.2% for the YTD.

PRICING

• In the China market 555 has consistently been able to retain a premium retail price to Marlboro and, therefore, more prestige.

• There was a shortening of supply of PM's brands to the market in the first half of 1992. It remains to be seen whether this has been a deliberate strategy following PM's increased sensitivity to exports having been chastised by the CNTC for supply irregularities or whether it has been the result of stock mismanagement. As a result, the price of Marlboro KS box (not the soft cup) showed an increase in the period of shortage to the premium level of that of 555 HKDNP stock, although the brand as a whole lost share. However, with the resumption of increased supply Marlboro's price is showing signs of slowly dropping back to its lower level.

SALES BY CHANNEL

• As CNTC outlets act as a showcase to Chinese smokers, BAT has ensured that 555's price is positioned at the premium end and above the free market price. This deliberate strategy is designed to enhance performance in the free market where the volume is significant. PM adopt a different approach, whereby Marlboro positioned in price both well below 555s price and below the free market price, has achieved brand leadership in the channel. Despite this, with three key brands BAT will still sell half as much again as PM, with a corporate share estimated at 27% in 1992 (compared to 18.8% for PM).

500014974

BATCo document for Lon Sch Hyg & Trop Med 12 February 2002

• Marlboro is also the largest selling brand in China Duty Free outlets (border shops, etc.) because of the brand's popularity outside China (e.g. Hong Kong).

COMMUNICATION SPEND AND AWARENESS

• Although BAT significantly increased communication spend in 1992, it is estimated that Philip Morris still spends as much behind Marlboro (more than US$20 million) as BAT for its four major brands (555, Kent, Lucky Strike and Hilton).

• With heavy support behind its global "Cowboy" campaign, PM has been able to achieve more awareness of Marlboro advertising than BAT for 555 and Kent (e.g. 88% for Marlboro in Guangzhou compared to 42% for 555 and 36% for Kent). Whilst advertising awareness for 555 has recently increased through outdoor and P.O.S. activities, with the introduction of the new international campaign, a further increase in advertising awareness may be anticipated.

• BAT's communications spend, although focussing on above-the-line, attach more weight than PM to outdoor and P.O.S. signages. As a result, 555 has a more competitive outdoor presence than Marlboro, the spend for which PM totally concentrate on electronic media.

ORGANISATION STRUCTURE

• A Philip Morris vice-president (W.S. Wong), based in Hong Kong, runs both the China domestic (CNTC and duty-free) and export businesses (as well as Taiwan). Wong reports to Bill Webb in Hong Kong.

• Philip Morris China Division has five sales offices in China and a small marketing department in Hong Kong. Both sales and marketing functions look after the domestic (CNTC and duty-free) business only. Philip Morris also has a regional marketing services department (also based in Hong Kong) to provide marketing support to all markets in the Asian region.

Information on performance, pricing and organisation structure are attached.

F. Chong
28 August 1992

500014975

BATCo document for Lon Sch Hyg & Trop Med 12 February 2002

SALES & SHARES

<< TOTAL CHINA >>	EST. 1992		1991		1990	
	Mn.	%	Mn.	%	Mn.	%
B.A.T.						
SE555	10942	16.3	8068	12.8	4571	10.5
KENT	5747	8.6	5652	9.0	4877	11.2
HILTON	16145	24.0	13894	22.0	9679	22.2
Others	3680	5.5	3310	5.2	519	1.2
TOTAL B.A.T.	36514	54.34	30924	49.0	19646	45.1
P.M.I.						
MARLBORO	9942	14.8	9720	15.4	5913	13.6
PARLIAMENT	222	0.3	99	0.2	78	0.2
MONTEREY	645	1.0	925	1.5	330	0.8
Others	543	0.5	139	0.2	130	0.3
TOTAL P.M.I.	11152	16.6	10883	17.2	6451	14.8
TOTAL CHINA	67200	100.0	63100	100.0	43539	100.0

21.08.92

500014976

BATCo document for Lon Sch Hyg & Trop Med 12 February 2002

SALES & SHARES

<< EXPORT >>	EST. 1992		1991		1990	
	Mn.	%	Mn.	%	Mn.	%
B.A.T.						
SE555	8674	15.3	6183	11.6	3085	8.8
KENT	5345	9.4	5216	9.7	4127	11.8
HILTON	15300	26.9	12836	24.0	8303	23.7
Others	3074	5.4	2820	5.3	259	0.7
TOTAL B.A.T.	32393	57.0	27055	50.6	15774	45.0
P.M.I.						
MARLBORO	7672	13.5	7540	14.1	3965	11.3
PARLIAMENT	114	0.2	44	0.1	78	0.2
MONTEREY	625	1.1	900	1.7	301	0.9
Others	341	0.6	135	0.2	130	0.4
TOTAL P.M.I.	8752	15.4	8619	16.1	4474	12.8
TOTAL EXPORT	56830	100.0	53430	100.0	35051	100.0

XIAMEN: CNTC	EST. 1992		1991		1990	
	Mn.	%	Mn.	%	Mn.	%
B.A.T.						
SE555	600	50.0	600	50.0	436	32.0
KENT					321	23.0
HILTON	600	50.0	600	50.0	609	45.0
Others						
TOTAL B.A.T.	1200	100.0	1200	100.0	1366	100.0
P.M.I.						
MARLBORO	-	-	-	-	-	-
PARLIAMENT	-	-	-	-	-	-
MONTEREY	-	-	-	-	-	-
Others	-	-	-	-	-	-
TOTAL P.M.I.	-	-	-	-	-	-
TOTAL XMCNTC/SEZ	1200	100.0	1200	100.0	1366	100.0

21.08.92

500014977

BATCo document for Lon Sch Hyg & Trop Med 12 February 2002

SALES & SHARES

<< CNTC >>	EST. 1992 Mn.	EST. 1992 %	1991 Mn.	1991 %	1990 Mn.	1990 %
B.A.T.						
SE555	685	13.0	429	9.3	240	8.4
KENT	120	2.3	157	3.4	120	4.2
HILTON	103	1.9	200	4.4	218	7.7
Others	516	9.8	408	8.9	169	5.9
TOTAL B.A.T.	1424	27.0	1194	26.0	747	26.2
P.M.I.						
MARLBORO	870	16.5	780	17.0	498	17.5
PARLIAMENT	100	1.9	53	1.1	-	-
MONTEREY	20	0.4	23	0.5	29	1.0
Others	-		-	-	-	-
TOTAL P.M.I.	990	18.8	856	18.6	527	18.5
TOTAL CNTC	5270	100.0	4590	100.0	2851	100.0

<< DUTY FREE >>	EST 1992 Mn.	EST 1992 %	1991 Mn.	1991 %	1990 Mn.	1990 %
B.A.T.						
SE555	983	25.2	856	22.1	810	19.0
KENT	282	7.2	279	7.2	309	7.2
HILTON	142	3.6	258	6.6	549	12.9
Others	90	2.4	82	2.1	91	2.1
TOTAL B.A.T.	1497	38.4	1475	38.0	1759	41.2
P.M.I.						
MARLBORO	1400	35.9	1400	36.1	1450	34.0
PARLIAMENT	8	0.2	2	0.1	-	-
MONTEREY	-	-	2	0.1	-	-
Others	2	0.1	4	0.1	-	-
TOTAL P.M.I.	1410	36.2	1403	36.3	1450	34.0
TOTAL D.F.	3900	100.0	3380	100.0	4270	100.0

21.08.1992

500014978

BATCo document for Lon Sch Hyg & Trop Med 12 February 2002

RETAIL PRICE PER 200'S

<< DUTY FREE >>	1992 HK$	1991 HK$	1990 HK$
B.A.T.			
SE555 K	70	70	65
KENT K SC	70	70	65
KENT K BOX	75	75	-
KENT DL SC	75	75	70
KENT DL BOX	75	-	-
HILTON K	40	40	35
HILTON SL	45	45	40
P.M.I.			
MARLBORO K	70	70	65
MARLBORO K BOX	70	70	-
MARLBORO 100S	75	75	70
PARLIAMENT 100S	75	75	70
MONTEREY 100S	40	40	-

<< CNTC >>	1992 FEC	1991 FEC	1990 FEC
B.A.T.			
SE555 K	68	66	60
KENT K SC	57	57	54
KENT K BOX	57	57	-
KENT DL SC	59	59	55
KENT DL BOX	59	-	-
HILTON K	38	33	36
HILTON SL	40	40	38
P.M.I.			
MARLBORO K	57	57	54
MARLBORO K BOX	57	57	-
MARLBORO 100S	59	59	55
PARLIAMENT 100S	59	59	55
MONTEREY 100S	36	36	33

GUANGZHOU << FREE MARKET >>	AUG'92 RMB	1991 RMB	1990 RMB
B.A.T.			
SE555 K	66	50	70
KENT K SC	53	49	55
KENT K BOX	53	50	-
KENT DL SC	50	48	58
KENT DL BOX	51	-	-
HILTON K	34	31	44
HILTON SL	34	32	44
P.M.I.			
MARLBORO K	64	58	59
MARLBORO K BOX	69	-	-
MARLBORO 100S	59	-	56
PARLIAMENT 100S	-	-	-
MONTEREY 100S	27		38

20.08 1992

500014979

BATCo document for Lon Sch Hyg & Trop Med 12 February 2002

LIST PRICE (HK$/CASE)

<< DUTY FREE >>	1992	1991	1990
B.A.T.			
SE555 K	2200	2050	1900
KENT K SC	2050	2050	1900
KENT K BOX	2050	2050	-
KENT DL SC	2100	2100	1950
KENT DL BOX	2100	-	-
HILTON K	1000	1000	950
HILTON SL	1050	1050	960
P.M.I.			
MARLBORO K	2050	2050	1900
MARLBORO K BOX	2050	2050	-
MARLBORO 100S	2100	2100	1950
PARLIAMENT 100S	2100	2100	1950
MONTEREY 100S	950	950	-

<< CNTC >>	1992	1991	1990
B.A.T.			
SE555 K	1500	1500	1500
KENT K SC	1300	1300	1300
KENT K BOX	1300	1300	-
KENT DL SC	1350	1350	1350
KENT DL BOX	1350	-	-
HILTON K	850	850	850
HILTON SL	900	900	900
P.M.I.			
MARLBORO K	1300	1300	1300
MARLBORO K BOX	1300	1300	-
MARLBORO 100S	1350	1350	1350
PARLIAMENT 100S	1350	1350	1350
MONTEREY 100S	850	850	850

XIAMEN: CNTC	1992	1991	1990
B.A.T.			
SE555 K	1950	1850	1850
KENT K SC	-	-	-
KENT K BOX	-	-	1550
KENT DL SC	-	-	-
KENT DL BOX	-	-	-
HILTON K	-	-	-
HILTON SL	1020	1000	1000
P.M.I.			
MARLBORO K	-	-	-
MARLBORO K BOX	-	-	-
MARLBORO 100S	-	-	-
PARLIAMENT 100S	-	-	-
MONTEREY 100S	-	-	-

<< EXPORT >>	1992	1991	1990
B.A.T.			
SE555 K	2000	1950	1950
KENT K SC	1700	1800	1800
KENT K BOX	1700	1800	-
KENT DL SC	1700	1850	1850
KENT DL BOX	1700	-	-
HILTON K	950	950	950
HILTON SL	1000	1000	1000
P.M.I.			
MARLBORO K	1950	1850	1800
MARLBORO K BOX	1950	1850	-
MARLBORO 100S	1950	1850	1800
PARLIAMENT 100S	1950	1850	1800
MONTEREY 100S	750	750	750

21.08.92

500014980

BATCo document for Lon Sch Hyg & Trop Med 12 February 2002

PHILIP MORRIS ASIA INC. - CHINA DIVISION

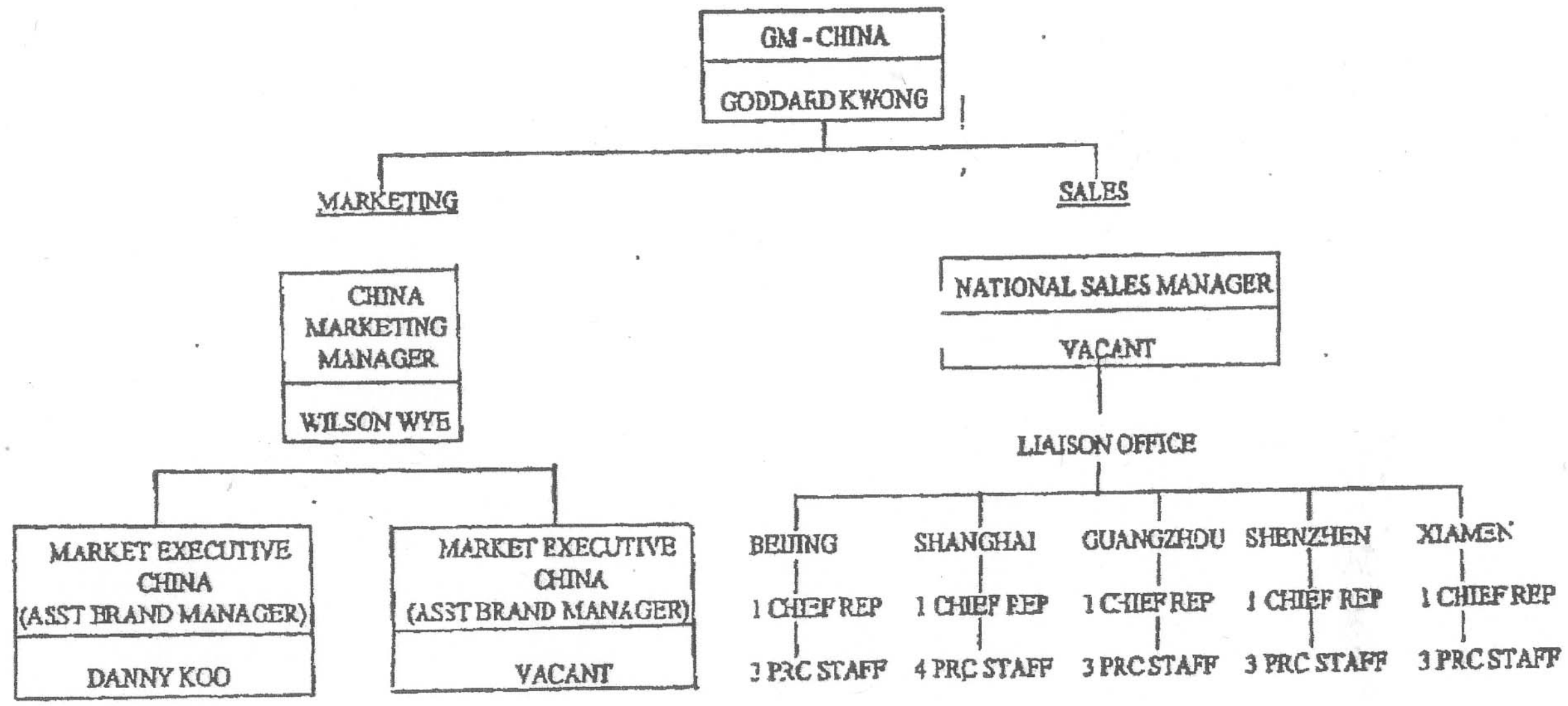

AUGUST 26, 1992

500014981

BATCo document for Lon Sch Hyg & Trop Med 12 February 2002

PHILIP MORRIS ASIA INC.

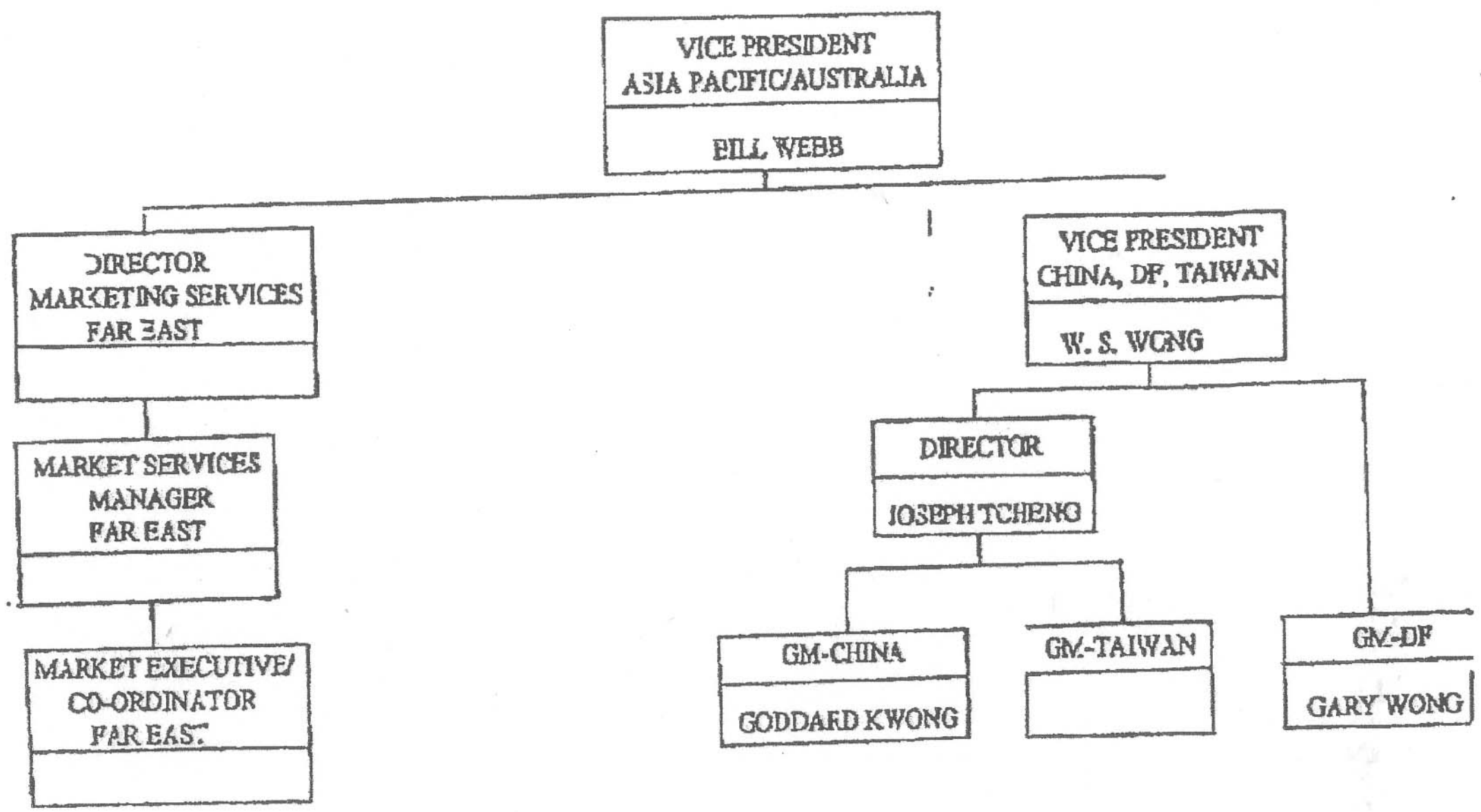

N.B. PMI RELY HEAVILY ON LEO BURNETT FOR MARKETING INPUT, PARTICULARLY SPEND PROPOSALS A-T-L, B-T-L

AUGUST 20, 1992

500014982

BATCo document for Lon Sch Hyg & Trop Med 12 February 2002
