# Supplementary material for: “Key to the Future”: British American Tobacco and Cigarette Smuggling in China
Source: PLoS Med. 2006 Jul 18;3(7):e228. doi: 10.1371/journal.pmed.0030228 (PMC1502159; doi:10.1371/journal.pmed.0030228)
Supplement: Supplementary file 8 [file pmed.0030228.sd008.pdf]

File - China Marketing.

BRITISH-AMERICAN TOBACCO COMPANY LIMITED

P.A.

1 SEP 1992

28 August 1992

SECRET

FACSIMILE

Mr T E Whitehair
Brown & Williamson Tobacco Corporation
P.O. Box 35090
Louisville
Kentucky 40232
U.S.A

Dear Tom,

by us all!

China Visit Notes

I attach a copy for you. We note your comment about the amount of work needed to be done behind KENT in that market. The biggest USIB issue for me is the free market pricing of LUCKY STRIKE which means that LUCKY STRIKE, VICEROY and HILTON compete at about the same free market price though each has regional pockets of strength. You explained in Millbank the difficulty of raising the price significantly without sucking in transit. I hope for the sake of the Group's only truly international brand the trade-off between strategic building of quality share in the market with the biggest potential growth and commercial, operational pragmatism can be successfully managed.

Agree - key point.

KENT's Positioning

We are not addressing semantics unfortunately. You explained to Jimmi and me your views in Millbank. You are the Brand Owner. When we re-issue the USIB Brand Plans after the TEC has approved the individual Company Plan budgets, we will state BWI's global positioning rather than the one we feel is appropriate for BATCo's markets.

BARCLAY's Age Profile

We note your comment and will apply it. We suggest that you and Bob look at the profile of the consumers of BARCLAY Ultra Lights in BATCo's European markets. We think that variant has opened up a new, younger group of consumers for the BARCLAY Family. If so, the brand's potential could be significantly widened.

REGD OFFICE MILLBANK KNOWLE GREEN STAINES TW18 1DY FAX 0784 455 000 TELEX 27384/5 BATTOB G
TELEPHONE 0784 460 400

A MEMBER OF THE BAT INDUSTRIES GROUP INCORPORATED IN LONDON No 74974

500014860

BATCo document for Lon Sch Hyg & Trop Med 12 February 2002

<u>LUCKY STRIKE & SONST NICHTS</u>

We note the contents of your fax to Rainer Grahn. We gather that the testing of your new "individualism" campaign to update "Biker" has not produced good enough results. For those BATCo markets currently using "Biker", we would appreciate knowing what the timetable is for new film and print pools? // Critical - we're desperate.

We agreed with you in Millbank to test Sonst Nichts outside sophisticated markets. Irrespective of the test results, we recognise that the Brand Owner obviously retains the right to decide what campaign is run by licensees. You suggested that we test in Indonesia which is probably our least advertising literate market. We have approached BATCF about that. We would also like to test in Venezuela to get a view in a virgin LUCKY STRIKE market where price parity with MARLBORO will be achieved.

We have an issue in Malaysia. MTC wishes to relaunch LSF at price parity to MARLBORO asap in 1993. MARLBORO is poised to explode. LSF product development is proceeding well. A cigarette print ban may happen 1/1/93. The only TV advertising allowed, as you know, is for TMD. MTC has developed an American Original retail concept but has had problems developing creative for it. We have suggested to BATCF that they and KNS could explain, for testing purposes only, the Sonst Nichts communications programme and adapt it for this TMD opportunity. Is this OK? Obviously BWI retains the right to decide what campaign accompanies the relaunch by this licensee.

Best wishes,

Iain

<u>I G HACKING</u>

cc: Mr J Rembiszewski
Mr P N Adams ✓
Mr J L Hendricks
Mr R J Miller
Mr Ooi Hoe Seung
Mr F Chong
Mr A A Chown

Attach.

500014861

BATCo document for Lon Sch Hyg & Trop Med 12 February 2002

SECRET

Visit Notes
BAT China
August 10 - 14th 1992

Objectives:

1. To learn about the performance and potential of the USIBs in China and address key issues with BAT China.
2. To make a first visit to China.
3. To review USIB communications issues with Grey.

Overall:

BAT China is a new entity. The Marketing Director has been in office barely two months. 3 months. The stance to the market is now marketing, and thus consumer, based after the trading entrepreneur mode of the previous administration which concentrated on building the volume base. A research database is being constructed. Tracking studies have commenced this year. Knowledge about the consumers of International Brands is increasing. Distribution improvements and the issue of pricing relativities are being addressed. It certainly appeared that the foundations of a marketing-led business have been laid and the marketing strategies refined. Connections and communications to Millbank Marketing were helped by this visit as the new BAT China management attempts to get more familiar in depth with BATCo's ways and practices.

BAT China has inherited a large and growing business. Hong Kong Exports of USIBs are 13% up at the half year compared with SPLY and on budget. The markets in Guangzhou and Shanghai were visited. The imported segment in Guangzhou is around 10% and Shanghai around 5%. Street presence in Guangzhou for cigarette brands is possible and 555 and KENT were highly visible. MARLBORO's street presence was negligible. I watched no Chinese TV. Good reach seems possible to achieve as $4000 for a prime time spot on CCTV can deliver an audience of 600 million viewers.

Key Issues:

1. **Is the US Brand business in good shape?**
The China Market Monitor indicates that BAT/B&W as a brand group is the clear market leader in China but that MARLBORO is the USIB brand leader in share and top-of-mind awareness. KENT is buoyant in Shanghai but losing share in Guangzhou and Beijing. LUCKY STRIKE's share in Guangzhou is flat and negligible elsewhere despite a doubling of shipments in the first half of 1992. VICEROY's share in Guangzhou has gone to 7% from 2%. Brand awareness has increased for the 3 BAT USIBs but so it has for competition as marketing activities are increased. Marl. is losing significant share.

The strategy which PMI appears to be following involves, as usual, focus on MARLBORO, heavy ATL investment, concentrating on the official retail outlets, and getting close to the CNTC officials. POS presence is negligible. In Shanghai which is KENT's stronghold PMI is spending behind PARLIAMENT following the same strategy as for MARLBORO. The CNTC stores are one small source for leakage into the free market. Thus PMI is pursuing a long term strategy to build awareness and brand values and maintain pricing at the highest level in the imported segment. | but losing share.

500014862

BATCo document for Lon Sch Hyg & Trop Med 12 February 2002

Despite the absence of historical consumer data it would seem that BAT's USIBs have a poorer quality of share than the clear leaders - 555 and MARLBORO. If, in this unsophisticated market, price largely equals image then KENT is at a disadvantage as it is clearly at one price point below the leaders. LUCKY STRIKE and VICEROY are sadly at around 50% of MARLBORO's price. HILTON is also at the same price and three Group brands priced similarly seems in portfolio terms to to be not optimal. In the short term, however, shipments of the Group's USIBs are growing rapidly.

What is the brand owners + central marketing point of view. As I understand it, they are responsible for the marketing mix.

ACTION: BAT China is aware of the issues and is constructing its 1993 Company Plan currently. This will be discussed in London in the Fourth Quarter.

2. **Can KENT achieve free market parity pricing with MARLBORO?**
It is only in 1992 that a first, truly marketing programme which was devised in late 1991 has been implemented for the brand. The brand appears to have been damaged by the pre-printed tipping issue, the over supply in 1991 and the asbestos rumour in late 1991. Some increase in retail price has been achieved in recent weeks as supply has been curtailed. BWI's research indicates that the consumer still views KENT in the premium price segment with 555 and MARLBORO. The launch of KS Box helped to restore KENT's volume. The aim of price parity is appropriate but in reality some discount to the leaders will probably need to be accepted.

So what should we do.

ACTION: BAT China will address this in its Company Plan.

3. **Is the KENT campaign effective?**
The Monitor indicates that KENT's unaided advertising awareness has declined in Shanghai, Beijing and Guangzhou in the current wave. Its level is far behind MARLBORO's. KENT's visual recall was significantly less than that for MARLBORO and 555 and in image-related mentions its recall was half that of MARLBORO's.

BWI has done 2 qualitative studies in China and both indicate that copy is not a problem.

Is this central marketing's P.O.V. Is the budget sufficient

ACTION: BWI will be testing the campaign again using animatics of possible new executions in September. Spending the media money effectively is being examined by BAT China.

4. **Can LSF's price be increased to near MARLBORO's price without catastrophic volume loss?**
LUCKY STRIKE is the Group's No. 1 priority international brand worldwide. Its free market price in China is far below the Brand Owner's strategic discount to MARLBORO. The PMI brand building strategy was not followed at launch and LSF is in the free market in Guangzhou where its price is half that of MARLBORO. The Brand Owner believes that any significant price increase will just suck in transit and that critical mass is needed before any major price repositioning can be embarked on. The Territorial Team and BAT China have agreed on a strategy which increases the marketing activity behind the brand and concentrates that activity on the Southern Coastal area. Consumer perceptions about the brand will be tracked. Price will be eased up.

500014863

BATCo document for Lon Sch Hyg & Trop Med 12 February 2002

If the price were raised to near MARLBORO now, the estimate was that half its annual volume - 500 million sticks - might be lost (though not all to competition). The GT price would also need to be controlled so the China LSF pricing decision could be near to a 1 billion stick decision. If the China market has the single biggest potential for BAT's brands of all world markets, the cost from a strategic viewpoint to put LUCKY STRIKE in a healthier position to pick up quality share growth seems affordable and necessary to the Group.

ACTION: The USIB Brand Group would like to conduct a study with Far East Territorial quantifying the financial implications and benefits of this action. The study should be ready by mid September.

5. **When will BAT China replace the USIB Marketing Manager?**
BAT China wishes to hire Chinese speakers with high FMCG skills and experience. Emigration has slimmed the size of available talent in Hong Kong. BAT China has been unlucky in its approaches to outside candidates. Its current strategy is to fill the two USIB brand management slots (the KENT brand manager recruited from the outside will start in mid October) and have the Marketing Director allocate more of his time to USIBs than UKIBs.

It is BAT China's aim to raise the responsibilities and calibre of its regional staff in China. By the start of 1993 BAT China should be in a position to improve the marketing of its USIB brands. Fortunately the consumer in China is still unsophisticated but competition in addition to PMI is now getting organised and starting to spend heavily in the market place.

ACTION: It is the responsibility of BAT China with Territorial endorsement to structure and staff its organisation. A sprinkling of expatriates in the Marketing Department could, however, facilitate the marketing of the USIBs despite the language problem and in these early stages might help to make communications with Millbank and other Group companies optimal. why?

6. **Does BAT China know what the USIB ATL budget is delivering?**
Not for KENT. The previous management committed most of the KENT ATL budget for 1992 and beyond. Two contractors or brokers have allocated the budget between TV, outdoor and bus signs in China. Each ATL buy was contracted separately and a large number of the contracts are binding for two to three years. An evaluation of what BAT China is getting for KENT is underway and an audit carried out by independent auditors will be implemented. Negotiations with the bigger supplier are in progress to assess what, if any, contracts could be assigned to Grey. This task is a high priority for the Marketing Director.

Most of the LSF A&P budget is available to BAT China and Grey.

ACTION: BAT China is in negotiations with the contractors and getting a grip on the status and opportunities is a key priority.

I G HACKING
28 August 1992

500014864

BATCo document for Lon Sch Hyg & Trop Med 12 February 2002
