# Supplementary material for: “Key to the Future”: British American Tobacco and Cigarette Smuggling in China
Source: PLoS Med. 2006 Jul 18;3(7):e228. doi: 10.1371/journal.pmed.0030228 (PMC1502159; doi:10.1371/journal.pmed.0030228)
Supplement: Supplementary file 9 [file pmed.0030228.sd009.pdf]

Copy Mr. P.N. Adams

A.A.C.
19 AUG 1992

2718

B.A.T. CHINA LIMITED

Inter-Office Memo

P.A.
28 AUG 1992

To : Frank Chong   From : Ng Kam Ka

Date : 17 August 1992

CC : See Distribution

File - China Marketing

Subject: **CHINA MARKET MONITOR 1992 WAVE II KEY FINDINGS**

Attached please find the above for your perusal. As this copy (dated 13 August 1992) precedes the report dated 3 August 1992; and to pre-empt any confusion, please shred the earlier copy accordingly.

Regards,

1. Ability of imports to grow at same pace
   - incidence among occasional smokers has dropped
2. Stay out [illegible] on box. Seems to have lost some steam + is still small in Beijing + Guangzhou
3. Menthol/Lights. small + static
4. 100s growing off minute base.
5. Brand awareness growing but not yet at Marl. levels
6. STT is losing in 3 cities. Where is it going? Should we increase monitor beyond 3 cities.
7. Adv. awareness low vs Marl + not really growing. STT - no strength in product + image rating

Marl. strong user image + strong product + user image in Beijing

Kent: image is declining

Distribution

B.A.T. China	B.A.T.Co - Staines
H.S. Ooi	Alan Chown
N. Lovett	Susan Osborne
C. Leung	Iain Hacking
H. Tso	
K.K. Wong	
C. Chan	

Joseph Wang - BSB
Leonie Ki - Grey Advertising

31ST FLOOR ONE PACIFIC PLACE 88 QUEENSWAY HONG KONG FAX: 877 6428 TELEX: 78237 BATCL HX
TEL: 514 2188

A MEMBER OF THE BRITISH-AMERICAN TOBACCO COMPANY GROUP INCORPORATED IN LONDON NUMBER 134409
REGD OFFICE: MILLBANK KNOWLE GREEN STAINES MIDDLESEX TW18 1DY UK

香港金鐘道88號太古廣場第壹座三十一樓 電話：514 2188 傳眞：877 6428 電傳：78237 BATCL HX

500014910

BATCo document for Lon Sch Hyg & Trop Med 12 February 2002

B.A.T. CHINA LIMITED

10 August 1992

Mr P. N. Adams
BATCo
Millbank, Staines
U.K.

Dear Paul,

Re : China Market Monitor Key Findings

Attached please find the above for your perusal.

As you are aware, we are conducting this quantitative CMM three times a year. However, in order to obtain an in-depth understanding of the consumer behaviour, mind set of smokers, and to identify and better understand smokers' language and vocabulary in China, we plan to conduct a smokers' usage and attitude qualitative study (I understand it has never been done before) at the end of August in three cities, namely Beijing, Shanghai and Guangzhou.

Based on the recent findings, a number of issues would need to be addressed :

I) SE555

The key issue obviously is the brand's image of SE555. Hopefully the new "Planet" approach would help to uplift the image, and would further enhance with the new campaign which is under development.

The improvement on the brand's image of Marlboro did not surprise us considered the amount of money PMI is putting behind the brand with a consistent campaign. Apart from not having a suitable campaign for SE555, we are also concerned about not providing enough communication support behind the brand.

Total Communication	1991 HK$mn	1992 HK$mn
SE555	28	75
Marlboro	140	Est. same as 1991 or higher

../2

31ST FLOOR ONE PACIFIC PLACE 88 QUEENSWAY HONG KONG FAX: 877 6428 TELEX: 78237 BATCL HX
TEL: 514 2188

A MEMBER OF THE BRITISH-AMERICAN TOBACCO COMPANY GROUP INCORPORATED IN LONDON NUMBER 134409
REGD OFFICE: MILLBANK KNOWLE GREEN STAINES MIDDLESEX TW18 1DY UK

香港金鐘道88號太古廣場第壹座三十一樓 電話：514 2188 傳眞：877 6428 電傳：78237 BATCL HX

500014911

BATCo document for Lon Sch Hyg & Trop Med 12 February 2002

SE555 sales volume is about 25-30% higher than Marlboro, but the level of support is very much lower. The brand has been selling based on its strength of heritage, which makes it very vulnerable. We could push as many cigarettes as possible, but to maintain a consistent growth in the long term would require a more strategic approach. We feel that we may be milking the cow, and not feeding it enough to maintain its premium positioning. We plan to address this issue in the 1993 Company Plan.

II) <u>Box Vs Soft Cup</u>

Another interesting finding is soft cup Vs box style. There is no doubt a demand on box in the market. However, we feel that at present it is a function of supply rather than consumer demand. This is further supported by the recent field sales report in Guangzhou that Kent soft cup is commanding a price premium to box in the wholesale and retail markets. If this continued, this phenomenon would eventually happen in other major cities as the availability of soft cup reduces. With the increase in the volume of box style, the price premium diminishes. This is one area we would have to monitor closely.

III) <u>Lucky Strike</u>

Due to its low price positioning, the brand is definitely suffering. We will explore the feasibility of implementing the strategy, i.e. drastic price increase as discussed with you, in 1993.

<u>Other Matters</u> :

1. <u>XMCNTC (SRBT)</u>

They have accepted our new pricing proposal effective January 1, 1993.

2. <u>China Duty Free</u>

They are still rejecting our new pricing proposal, but we plan to stick to it. The only concern is PMI may give in to CNDF pressure and continue to supply Marlboro at old price. However, we will continue to monitor the situation.

Best Regards,

Ooi Hoe Seong

500014912

BATCo document for Lon Sch Hyg & Trop Med 12 February 2002
