# Supplementary material for: “Key to the Future”: British American Tobacco and Cigarette Smuggling in China
Source: PLoS Med. 2006 Jul 18;3(7):e228. doi: 10.1371/journal.pmed.0030228 (PMC1502159; doi:10.1371/journal.pmed.0030228)
Supplement: Supplementary file 10 [file pmed.0030228.sd010.pdf]

– Means of leakage?
– How do we keep fair to other exporters?
– Test list
– They need to be CNTC blessed.
– Need clear contractual commitments
– nothing vague and U/V to expectations.

— Merchandising and branch — E.S two major ones? vs Inchcape + Jardines
Att P.N.A.
Paul, just a 1st draft. Can we have your comments, please

File - China Marketing

DISTRIBUTION INITIATIVES WITHIN PRC

Background

One of BAT China's key objectives is to improve distribution of BAT brands within China, and thereby the availability of the product to the consumer. Initially and primarily this objective is focussed on officially imported duty paid CNTC marked stock, but as long as free market sales remain dominant, alternative routes of distribution of unofficial imports need to be examined, evaluated and, if appropriate, maximised. It is recognised that distribution of our product in China is key to BATCo's long term success and a key objective within BAT China's JV initiative is to achieve widespread distribution networks.

Official distribution of duty paid imported product is exclusively controlled through the state monopoly, CNTC, and, so far, no other company may be involved with this function. The distribution system for officially imported duty paid product is therefore weak, favours local brands, is often reliant upon forex allocations, and relies almost entirely upon the retail trade to collect stocks from CNTC warehouses, although some satellite wholesale areas are emerging in some of the larger cities. Virtually all duty paid cigarette imports are channelled through agents appointed by the individual CNTC branches, who arrange logistics and interface with the suppliers, in return for a commission. Originally, the function of these appointed agents was to actively seek orders amongst the CNTC branches, but in recent years there role is nothing more than order takers, and to provide commission / gratis collection and PR services on behalf of the individual CNTC branch.

A number of non-tobacco international fmcg companies are moving to quickly enhance distribution of their products through established trading companies like Jardines, Inchcape, Swires etc. however, in the short term at least, such networks are not available to international cigarette companies. Nevertheless, all opportunities need be explored aggressively, even if any initial co-operation with an established distributor covered only merchandising and/or delivery of official duty paid import product to 'official retail outlets'.

Key competition will obviously be seeking the same objectives, and there are strong indications that PMI have already appointed 'distributors or agents' in certain provinces within China to act on their behalf, that would actively interface with CNTC Beijing and branches, although details of such arrangements are extremely sketchy. Reports suggest that Goldpoly, PMI's exporter for the China market, have been granted this right for defined provinces, with the support of PMI and endorsed by CNTC, but we have no concrete evidence to confirm such arrangements. It is important that BAT do not get left behind. ?

Possible Distribution Scenario within next 3 years

Free markets will continue to dominate, supplied through current export sources, enhanced by the growing influence of leakage from duty free points throughout China. This latter channel will help to improve the overall spread of penetration rather than the historical reliance on exporters landing stock in southern areas, where profits can be realised that much easier and quicker, rather than the risk/expense of sending stocks further north. Additionally, however, it could well be that provincial entities of CNTC will seek to increase their own leakages into free markets, of officially imported CNTC marked (CM stock), in order to maximise revenue and profitability. It should also be recognised that this CM stock may have increasing trade and consumer attraction if counterfeit continues to be a problem, as well as the withdrawal of SEFK hkdnp marking. ?

To protect exporters long term business interests in a product group that provides substantial profits and cash, the major export customers will be conscious of the need to add value by providing improved services to their principal suppliers, particularly as they become aware of the growing influence of duty free channels and use of overland routes. Given the nature, wide sphere and high level of influence, even within PRC, of these main exporters used by both BAT and PMI, they (primarily Goldpoly, and Eternal State - Giant Island do not appear to be at all proactive in this area)

500014779

BATCO CONFIDENTIAL - CATEGORY I: MINNESOTA TOBACCO LITIGATION.

BATCo document for Lon Sch Hyg & Trop Med 12 February 2002

will be keen to cement their business relationships with their main suppliers and to move towards legitamizing their business as quickly and effectively as possible.

Consequently, exporters have already and will continue to seek to establish their own representative offices in key defined cities, to oversee a wide variety of activities and investments, not just tobacco related. Specifically allied to tobacco, our exporters will seek close alliances with specific local CNTC networks. It is therefore likely that provincial CNTC offices will be persuaded to appoint these exporters as their official agents for importing officially duty paid import stocks, as is currently done by virtually each CNTC branch for official duty paid imports. BAT China has dealt with a variety of such representatives, for some years now, with most of the appointees made during the time of Wei Pong and Leo Chan. Having 'legitamised' their operations with CNTC, as officially appointed agents these exporters like Eternal State will then be able to serve the purpose for which they were originally intended; to actively seek orders from key provincial CNTC offices, but more importantly exporters like Eternal State would provide finance to the local CNTC authority to enable them to purchase and distribute officially imported stock.

This will also involve the establishment of exporters' own networks within PRC, which, whilst not fully fledged distribution set-ups, will have a strong influence on distribition through the alliances formed with provincial CNTC branches. Any resulting profits could well be re-channnelled into other non related local operations and investments that each exporter already has in place in PRC.

Recommendation

Eternal State (Wing Lee) have been highly proactive in wanting to establish their own networks within China, and advise us that they have already made representations to CNTC Beijing and other CNTC branches. They have asked for BAT's support in exploring this further, which has been given verbally, as well as a broadly worded letter, with no commitments, stating that they have BAT's blessing to explore such initiatives with CNTC, provided of course that such initiatives have the full support of CNTC. Any such letter would be no different than a recommendation from BAT to CNTC that they consider use of Eternal State (or associate company) representing CNTC's import interests, in the same way that BAT China use the myriad of CNTC appointed agents for our current CNTC business.

Does ES, understand it this way??

We would want to insist that any such arrangements were exclusive to BAT products which would need to be monitored, although it is doubtful that BAT would want to commit to any formal agreement, at least in the short term. One advantage that the exporter with whom we co-operate would seek is that that exporter receives incremental export product allocations, at the expense of other exporters who are unable to provide such a competitive 'distribution' service. BAT would benefit through a wider spread of distribution, particularly in secondary and tertiary cities, ahead of competition, and an increased core CNTC share, which is currently under threat.

It is evisaged that this initiative would be a short to medium term strategy prior to establishment of any JV operation and naturally any action taken would need to run in parallel with our JV plans as well as alongside our own exploration of official distribution potential through other established third parties (eg JDH, Jardines, Swires) if current legislation was relaxed.

very impt.

very important

<u>This proposal would only be progressed further if original documentary proof can be provided from CNTC and separately confirmed by BAT China with CNTC that there is universal agreement to such arrangements.</u>

who manages this in China - BAT China

If this is forthcoming, and confirmed as entirely official, BAT should allow them the opportunity of a test province to work.

NAL
18/11/93

500014780

BATCo document for Lon Sch Hyg & Trop Med 12 February 2002
