# Supplementary material for: “Key to the Future”: British American Tobacco and Cigarette Smuggling in China
Source: PLoS Med. 2006 Jul 18;3(7):e228. doi: 10.1371/journal.pmed.0030228 (PMC1502159; doi:10.1371/journal.pmed.0030228)
Supplement: Supplementary file 11 [file pmed.0030228.sd011.pdf]

247

BRITISH-AMERICAN TOBACCO COMPANY LIMITED

19 August 1993

SECRET

Mr. U. Herter,
C/o UTICO Holdings Limited,
Johannesburg.

Dear Ulrich,

RE: CHINA - JV'S

Further to my fax to you yesterday, I thought it me be helpful to summarise the Asia Pacific RBU stance vs that of PMI.

The details of the PMI deal are still unclear but the essentials appear to be:-

1. Contract/licensed manufacture of MARLBORO, RED & WHITE and a joint brand.

2. "Immediate" manufacture of MARLBORO (i.e. sometime next year).

3. Commitment to export RED & WHITE to CIS and Eastern Europe.

4. Cooperation on production, leaf processing operations and product development.

The Asia Pacific RBU have recommended that we:

1. Pursue majority owned J.V. first, with fall-back of contact/licensed manufacture.

REGD OFFICE MILLBANK KNOWLE GREEN STAINES TW18 1DY FAX 0784 455 000 TELEX 2736475 BATTOB G
TELEPHONE 0784 460 400

A MEMBER OF THE BAT INDUSTRIES GROUP INCORPORATED IN LONDON No 74474

500015980

BATCo document for Lon Sch Hyg & Trop Med 1 February 2002

2. Avoid commitment to large scale production of international brands immediately (e.g. 555 and KENT).

    - this would be subject to:-

        - experience of satisfactory quality production

        - consumer acceptance of local production

3. Avoid export commitments (learnings from Pecs acquisition).

4. Be prepared to include leaf, training, and product development in cooperation.

We believe that the J.V. route is the better option as it provides:-

1. Management control

    - essential for control over quality, brands and people (i.e. right to hire and fire)

2. Greater capability to expand.

3. Share of manufacturing profit.

4. A stronger, long-term partnership.

Our real concern is that with a licensed operation, particularly in a Chinese environment, local manufactured quality would suffer on a long-term basis. This is not the way to build the business in China.

Inevitably there are some risks to us:

1. Philip Morris may encourage a G.T. clamp-down.

2. J.V. negotiations would at best be lengthy (historic experience) and not necessarily successful given the CNTC declared reluctance to enter any more.

However, we believe we should give ourselves a limited time to vigorously explore this route.

500015981

BATCo document for Lon Sch Hyg & Trop Med 1 February 2002

Philip Morris also have risks.

1. They are exposing their local produced MARLBORO to rejection by consumers, or at least a significant discount (witness the price at which China-claused stock sold in 1992).

2. Product quality may well suffer.

3. Financially and strategically not the best term option.

I would be happy to discuss this with you further.

With kind regards,
Yours sincerely,

Paul

P. N. ADAMS

c.c. Mr. B. D. Bramley
Mr. N. Davis
Mr. R. H. Pilbeam
Mr. J. Rembiszewski
Mr. R. M. H. Duncan
Mr. H. S. Ooi (BAT China)

500015982

BATCo document for Lon Sch Hyg & Trop Med 1 February 2002
