# Supplementary material for: “Key to the Future”: British American Tobacco and Cigarette Smuggling in China
Source: PLoS Med. 2006 Jul 18;3(7):e228. doi: 10.1371/journal.pmed.0030228 (PMC1502159; doi:10.1371/journal.pmed.0030228)
Supplement: Supplementary file 12 [file pmed.0030228.sd012.pdf]

29 June 1994

Mr. R. M. H. Duncan
Mr. H. S. Ooi
Mr. D. S. Jones
Mr. G. Li

<u>CHINA - J.V.</u>

Barry Bramley and I were invited by Martin Broughton to a dinner last Thursday night for the Chinese Ambassador to London, Mr. Ma Yuzhen. Eagle Star were also there.

During dinner we talked the Ambassador through our desire for an equity j.v. in China. He said that China was keen to protect the profits of its tobacco business. We said that China's profits would be <u>greater</u> if they had a j. v. with us. The reasons given for this were:-

1. Demand for our international brands outstrips the supply via the CNTC Friendship Stores and Duty Free shops. This created a black market. Substantial domestic manufacture by a j.v. would provide China with a share in the profits hitherto taken by smugglers.

2. Chinese consumers were up-trading to international brands which have substantial premiums. A share in higher margin international brands could be as lucrative as the total take on low margin domestic brands.

3. It would be better to j.v. with us now, when the market was protected and denied to us in totality, than wait until GATT provided access and we did not need a j. v. as much.

4. We have had a long relationship with China and have and are providing significant technology transfer. The one area so far missed by China was the "intellectual" technology of marketing and sales. This we could bring.

5. If BAT had a substantial presence in China it would improve the quality of leaf growing and enhance export potential for leaf. Additionally, it would attract the local manufacture of WMS by international companies, <u>producing to an international standard</u>, which in turn could lead to significant export opportunities. Both of these are illustrated by what has happened in Brazil where leaf and WMS are major exports.

In short China had much <u>more</u> to gain with us than without us.

502616630

JUN 30 '94 10:42AM B.A.T. CHINA LTD 852 877 5428 B.A.T. CHINA LTD P.3

BATCo document for Lon Sch Hyg & Trop Med 12 April 2002

He said he found the arguments persuasive and asked for a paper to be sent to him, re-iterating these arguments, and he would be pleased to have it forwarded it on - presumably to Beijing.

Barry and Martin are keen that we do so and quickly Please would you develop this paper within the next two weeks.

Some thoughts:-

1. I believe the paper should be brief and pithy - about two pages - otherwise it will not be read.

   However the points will need substantiation and illustration. This should be contained in an appendix, I suggest.

2. We should look to other BATCo. j. v.'s which have benefited the former Monopoly, e.g. Spain. David Bishop may be able to help.

3. We need some numeric examples to illustrate the points.

4. We should make it quite clear that we are looking for a substantial j.v. that is of an economic size, i.e. at least 15 billion production capacity and preferably nearer 40 billion.

5. Are there any other points that should be made?

Once you have had a chance to digest this to discuss forward action and timing.

P. N. ADAMS

c.c. Mr. B. D. Bramley
Mr. B. J. Kemball
Mr. P. C. O'Keeffe
Mr. R. S. Cameron

502616631

JUN 30 '94 10:42AM B.A.T. CHINA LTD 552 877 6428 B.A.T. CHINA LTD P.4

BATCo document for Lon Sch Hyg & Trop Med 12 April 2002
