# Supplementary material for: “Key to the Future”: British American Tobacco and Cigarette Smuggling in China
Source: PLoS Med. 2006 Jul 18;3(7):e228. doi: 10.1371/journal.pmed.0030228 (PMC1502159; doi:10.1371/journal.pmed.0030228)
Supplement: Supplementary file 13 [file pmed.0030228.sd014.pdf]

摘要

背景
香烟走私是一个影响公众健康的主要问题, 它刺激烟草消费的增加而且影响烟草控制措施的实施。中国是烟草消费量增长最大的市场, 也被确认为是世界上香烟走私最严重的国家。以前的工作已经证明,英美烟草公司（以下称BAT）在亚洲和前苏联地区内打击这一非法贸易活动的复杂性。

方法和调查结果
本文分析了一些 BAT 的内部文件。这些文件来自英国 Guildford 的存档处和 BAT 网站的文件库。一个专门设计的数据库,针对从二十世纪初到 2003 年的文件进行了搜索和编目，以此对历史作了描述。在文件解释过程中使用了多种确认技术。本文还描述了中国香烟走私的巨大规模，其总价值达几十亿美元;以及中国香烟走私的主要路径。并且研讨了 BAT 为获取最大利润，在更改操作结构，控制供应渠道和走私香烟订价的方面所作的种种努力。

结论
本文显示了走私在 BAT 打入中国市场的不断努力中所起的关键战略作用，BAT 的最终目标是在这一日益国际化的产业中取得主导地位。这些结果表明有必要加强全球性的努力来反对香烟走私。
