# Supplementary material for: “Key to the Future”: British American Tobacco and Cigarette Smuggling in China
Source: PLoS Med. 2006 Jul 18;3(7):e228. doi: 10.1371/journal.pmed.0030228 (PMC1502159; doi:10.1371/journal.pmed.0030228)
Supplement: Supplementary file 14 [file pmed.0030228.sd013.doc]

**“Key to the future”: British American Tobacco and cigarette smuggling in China**

***The Hong Kong-China supply route***

Hong Kong has provided the most important route into China for contraband cigarettes from the early 1980s when the territory was still under British colonial rule. Documents describe billions of sticks shipped by BAT UK and Export (BATUKE) and US subsidiary Brown & Williamson to either BAT Hong Kong (BATHK) directly or via Singapore depending on the brand and place of manufacture. The initial motivations to do so were to take advantage of a highly lucrative trade, and the belief that competing companies would do so if BAT did not:

*Frequent transit sales opportunities to transit markets arise in Hong Kong and BAT HK’s refusal to supply will not necessarily force the transiteers to seek supply from another BAT Group company designated to supply to that particular transit market. On the contrary, this will force the transiteers to purchase competitive products in Hong Kong in order not to miss the transit sales opportunities* [^[1]^].

Figure S1 illustrates these supply routes in 1991. For example, the brands SE555 and Benson & Hedges were shipped by BATUKE to BATHK. A more complex arrangement is described for the Hilton brand which was manufactured under license from B&W by BATHK. Hilton was also manufactured under contract to BATHK by STC and then shipped to either Hong Kong or Singapore. Of particular significance is the note in Figure S1 describing how cigarettes in Singapore are “handled by SUTL [Singapura United Tobacco Limited] as agent”. SUTL was an established distribution partner of BAT and did handle legal sales of BAT products, but documents describe that SUTL was also effectively granted a license to oversee smuggling on BAT’s behalf throughout much of Asia [^[2]^]. The mission statement for SUTL’s distribution strategy 1995–99, for example, was defined as

*To maximise BAT’s market and profit shares of the Southeast/Asia/Indian Sub-Continent export business through the most efficient distribution of international and regional brands, irrespective of sub-channel (Domestic, Duty Free, GT ) to our customers* [ ^[3]^].

GT designates “general trade”, perhaps the most frequently used euphemism for contraband operations in Asia [^[4]^,^[5]^], and GT cigarettes were then sold on by BAT HK to “exporters” for illegal import into the Chinese mainland. The role of “exporters” in handling GT is made clear in Figure S1 by their juxtaposition with legal imports via CNTC and duty free sales. Further evidence of the illicit nature of this trade is a raid in 1994 by Hong Kong authorities of one such exporter, Giant Island Limited. It was alleged that the company had bought HK$5.36 billion (US$694 million) worth of cigarettes from BATHK, and had smuggled 16 billion BAT cigarettes into China that year [^[6]^]. Similarly, in the prosecution and conviction of BAT HK export director Jerry Lui for smuggling and corruption, Mr Justice Wally Yeung Chun-kuen stated that “"The evidence suggests that management of BAT (HK) was aware duty-not-paid cigarettes ... would ultimately be smuggled in China and other countries. There could be no other explanation for this enormous quantity of duty-not-paid cigarettes worth billions and billions of [Hong Kong] dollars” [^[7]^].

Procedures to minimise paper records, maintaining an arms length relationship between BAT and distributors, were detailed by BATCo Project Adviser Asia/Pacific David Leach:

*There is no documentation for sales by BAT to the exporters other than export documentation comprising: -*

- *letter issued by exporter for cargo receipt*
- *export permit (in triplicate)*
- *BAT invoice printed at warehouse*
- *“picking list”*
- *confirmation of receipt by carrier*

*Sales to exporters in H.K. are on a cash basis, and are conducted in a matter of hours* [ ^[8]^].

This enabled BAT to disclaim responsibility for what happened to the cigarettes once legally sold to distributors. However, other documents indicate awareness by BAT staff that these shipments were intended for the contraband market:

*5. Following the reintroduction of B.A.T. cigarette products into the Peoples [sic] Republic of China, production of supplies for this market was commenced in U.K. The cigarettes were then, and are now, forwarded to B.A.T. Co. (Hong Kong) Ltd., for warehousing prior to call forward, by order, to outlets in China.*

*6. The cigarettes produced in U.K. for China are State Express 555, Product Code 5922, i.e.* ***GT [general trade]*** *unmarked stock, bar coded, and at all stages of their movement to China are Duty Free [bold added]* [^[9]^].

A “relaxed” [^[10]^] attitude by customs officials was sometimes relied upon, as well as a lack of communication between customs and excise officials in Hong Kong and China. Improved liaison between China and Hong Kong authorities after the handover of the territory to Chinese rule in 1997 was anticipated as a “problem” [Error: Reference source not found], as was the somewhat ironic perception of failure by Chinese authorities to follow “proper legal basis” in recognising BAT’s claim of limited liability if contraband was seized:

*PRC little concept of limited liability of company. Attitude that responsibility of Group for actions of subsidiaries including representative offices, and employees. As there is no extradition treaty between PRC and HK, PRC has simply arrested local representatives or officer within PRC to answer charges* [Error: Reference source not found].

The true scale of the illicit trade between BATHK and China is difficult to assess accurately given data limitations. A volume analysis by BAT’s Iain Milne, however, suggested that in 1992 “Hong Kong exports” by BATHK totalled 33 997 million sticks. While a small proportion of these exports were destined for other markets (e.g. Macau), the vast majority (90%) were for the illegal Chinese market, dwarfing BAT China’s legal imports of 4 312 million sticks that year [^[11]^].

***Supplying the northern and central provinces***

While the key mainland markets for contraband cigarettes were major cities such as Shanghai, Guangzhou and Beijing, substantial amounts were transported onwards to northern and central provinces. These regions were viewed as critical for future sales growth, BAT’s aim being “TO INVESTIGATE ALTERNATIVE EXPORT ROUTES/CUSTOMERS THAT WILL IMPROVE PENETRATION OF UK BRANDS IN NORTHERN AND CENTRAL PROVINCES” [^[12]^]. The complex supply routes to these regions also consisted of contraband smuggled overland from Russia and central Asia. This trade arose amid “PRC ENFORCED MOVES TO HALT GTM [general trade market]”, leading the company to “CREATE NEW/IMPROVED ROUTES INTO CHINA” [^[13]^], As BAT established a greater presence in the former Soviet Union [^[14]^], documents suggest that overland supply routes began to be used to transport B&W’s US international brands (USIBs). For example, Figure S2 shows how mid-priced brand Kent manufactured in Macon, Georgia in the U.S., was shipped to Russia and then transported to Kazakhstan for overland transport east to China in apparent quantities that dwarf stock destined for Turkey and Iran (see Figure S2).

Documents describe the role of other BAT subsidiaries in seeking to direct the region’s transit trade:

*It is known that business in this region is a transit business. The markets are supplied from various sources like Russia (Moscow traders), Belarus, Georgia and Turkey/Levant. At present, B + W are working these markets from their Istanbul office, run by Eric van Duyn, and BATCF [BAT Germany] are working out of the Sofia office, run by Boris Ivanov. It is recognized that given their limited management resources, neither B + W nor BATCF can exploit the potential in these markets….with a better infrastructure Group sales could reach up to 1.5 bns [billions] in 1995* [^[15]^].

These areas were also supplied by so-called “leakage” from the Sino-Russian Border Trade (SRBT). The SRBT formed part of BAT’s legal exports to China, arriving in the port city of Xiamen from BATHK with duty free status for onward barter with Russia [^[16]^]. While the legality of this border trade is unclear, the documents show BAT staff willing to examine “the possibilities of an alternative supply route to meet genuine SEFK [State Express Filter Kings] military demand on the Sino-Soviet border” [^[17]^]. Concerns about irregularities in the supply chain, from export from BAT factories to the point of sale to consumers, were raised in 1983 by a BATHK delegation to Beijing to negotiate an extension of a Consignment Contract. John Payne (BAT Group Advisor on Chinese Affairs) wrote of “the leakage of cigarettes from the People’s Republic of China (PRC) to other Far Eastern territories” [^[18]^]. By the mid 1980s, the large quantities of cigarettes going astray from this trade led BAT officials to suspend supplies to Xiamen pending further investigation. Internal inquiries revealed that the company was not wholly in control of the supply chain in China [^[19]^]. The need to reassert such control was important for protecting a third key supply route into China - Singapore.

***The Singapore-China connection***

An inability to exert full control over subsidiaries and distributors proved a growing problem for BAT. In 1984, suspicions within BAT emerged that “cigarettes destined for Duty Free markets in China were being diverted and supplied from Singapore to Bangladesh by a local transiteer,” referred to within BAT as “leakage” or “parallel imports” [Error: Reference source not found]. Complaints “reached a crescendo” in 1985 when Tay Choon Hye, chairman of key distribution agent SUTL, found stock of UK manufactured cigarettes not bearing the code “peculiar to his operation”. Given exclusive rights by BAT to supply the contraband market in Bangladesh and neighbouring countries, Tay objected to such “business competition” [Error: Reference source not found]. He reported that “[t]he speed of parallel shipments has been accelerating and he is extremely concerned that the intervention of **another transit party** could damage the entire business” (bold added). To mollify Tay, BATUKE gave a “5% discount specifically on his SEFK sales to Bangladesh in the month of December” which was seen as “a modest cost” [^[20]^].

As so-called parallel imports by third parties unauthorised by BAT continued, the BAT Group Securities Department identified as possible culprits “Hong Kong and Singapore business personalities who had come to notice on previous occasions as transit agents” [Error: Reference source not found]. Shipping documentation and export permits revealed five container loads of cigarettes exported from Hong Kong under five different names but all of which “gave the same contact telephone number in Hong Kong” of Leung Yick Company.

In August 1986, Mark Aitken (BATHK), Wai Pong (BATHK) and Ronayn Coburn (BAT Singapore) met in Hong Kong to discuss suspected leakage. Given a substantial escalation of shipments of SEFK to the Chinese port city of Xiamen [^[21]^], it was felt that this was probably the "sole source of China leakage in recent months" and agreed supplies should be stopped temporarily [^[22]^]. A further meeting was held in September 1986 to discuss measures to "prevent further leakages of SEFK from China/Hong Kong into other markets, notably those under the control of SUTL". Coburn writes in his notes on the meeting,

*[I]t was yet again made clear to BATHK that the continued disruption to BATUKE/SUTL interests in the key Bangladesh market resultant from massive leakage from Hong Kong was totally unacceptable. This situation, it was reiterated, was not only putting the BATUKE/SUTL operation and relationship at increasingly serious risk but also, in Group terms, placing in grave jeopardy a source of major financial contribution.*

*In these circumstances BATUKE would have no hesitation in resorting to draconian measures in order to impose control, accepting the possibility that this action could result in some loss of genuine business, in the belief that the preservation of the SUTL custom is of greater moment…*[Error: Reference source not found].

Another potential source of leakage was Taiwan, where Coburn noted “*a significant increase in demand from Taiwan for transit supply, in particular for MARLBORO and to a lesser extent, SEFK”* [Error: Reference source not found]. It was suggested that this could be another attempt to divert stocks of SEFK to Singapore and Bangladesh, rather than a reflection of increased demand in Taiwan: "In the circumstances it was agreed that SEFK transit supply [to Taiwan] should be monitored with extreme care.…" [Error: Reference source not found].

Such concerns led to the suspension of supplies to Xiamen and Shenzhen (35 kilometres from Hong Kong) in October 1986, with trade subsequently resuming under a warning that future “[l]eakage of any quantity, even one case, will trigger immediate cessation of this business” [^[23]^]. This resumption of supplies was largely motivated by a concern to keep pace with the availability of competing brand, Marlboro:

*If the Xiamen bit is genuine, then something else is not. I quite agree that we should have shut off the escalating Ships Stores business out of Hong Kong, but it was in the context of increasing Taiwan transit demand, where Marlboro has gone through the roof.*

*The problem with Shenzhen is that they are cheating, but again how much? … Our only ultimate solution would be to cease supplying them with SEFK completely, but with this scenario they would probably cease to buy any of our other brands, amounting to 50 mns [million sticks] per month…*[Error: Reference source not found].

Tighter control of supplies to Xiamen entailed the development of a closer relationship with the Chinese army, with deliveries and train loading receipts to be monitored [Error: Reference source not found]. While BATUKE’s Brian Brockman expressed doubt about the value of the Xiamen trade, regarding it “in the main ‘substitutional’ business and of dubious commercial merit" [Error: Reference source not found], Aitken was convinced of its potential significance:

*[W]e have never doubted the existence of a demand on the Sino-Russian border. As a result of one important new circumstance, i.e. meeting and talking direct to the Army, we believe, and we hope we are right, that the business is genuine. In that belief it would be wrong to shut it out, or at least not try and prove conclusively the validity of that belief* [Error: Reference source not found].

By 1987 the Xiamen trade seemed to be running more smoothly for BAT and it was reported that “[d]istribution expansion in China to the Sino-Soviet border areas prompted good growth to this important market” [^[24]^]. Improved control of the trade was assisted by the introduction of special coding to track stock intended for specific markets. As noted in a telex from Wai Pong, BAT HK:

*I note that a meeting with military scheduled in Xiamen this week to discuss brand allocation which U believe will lead to SEFK issues being reduced to approx 29 mns monthly. Until we know the outcome of the meeting the supply of ZM code should continue to be restricted to Xiamen.*

*If however yr assumption of an imminent reduction in Xiamen SEFK offtake is correct I feel the follwg policy should be adopted on issues/codes in future. ZM - continue as policy to restrict issues of this code to Xiamen as by so doing any leakage can be identified without argument…*[^[25]^].

By 1989, sales of SEFK reached 54 million sticks per month and Kent was expected to reach 180 million sticks [^[26]^], facilitated by the use of a careful system of coding:

*Sales to the Sino-Russian Border Trade for the month reached 54 Mn [million]. BAT UK&E has agreed to supply a specially coded SEFK stock of 40 Mn per month to this outlet. Upon agreement by Brown & Williamson for supply of Kent to this trade, the sales to this outlet can start in March and the volume may reach 180 Mn per month* [^[27]^].

Similarly, documents describe how SEFK destined for China (with a cited annual volume of 24 000 million sticks) was given a “substitute” product code 6623 “to identify transit into Taiwan” [^[28]^].

**Figure S1.** Contraband Supply Route to “Exporters” via BATHK

**Figure S2.** Contraband Supply Route of Brown and Williamson International Brands to China via Central Asia and Caucasus

1. . Wai Pong. NOTE FOR GM, TRANSIT SALES. 21 Nov 1984. BAT Hong Kong. Bates No. 301742777. <http://bat.library.ucsf.edu/data/o/q/p/oqp01a99/oqp01a99.pdf>
2. . Ross IA. SECRET, PROPOSED RE-DEFINITION OF MARKET RESPONSIBILITIES. 16 Dec 1988. BAT Singapore. Bates No. 500014649-4660. Available from Guildford Depository (Accessed 10 Jan 2005).
3. . Coombe F. SUTL Distribution Strategy 1995-1999. Aug 1994. Bates No. 500045730-5752. Available from Minnesota Depository (Accessed 15 July 2003).
4. . BATUKE. Company Plan, 1991-1995, Part 1. 10 Oct 1990. Bates No. 301615548-5583. Available from Guildford Depository (Accessed 10 May 2003).
5. . Collin J, LeGresley E, MacKenzie R, Lawrence S, Lee K (2004) Complicity in contraband: British American Tobacco and cigarette smuggling in Asia. Tobacco Control 13 (Supplement II): ii104-ii111.
6. . Campbell D (2000) Note of evidence by Duncan Campbell in respect of planning, organisation and management of cigarette smuggling by British American Tobacco Plc, and related issues. London: UK House of Commons Select Committee on Health, Inquiry into the Tobacco Industry and the Health Risks of Smoking, 14 February. Available: [www.gn.apc.org/duncan/bat/Health_Committee_Evidence_1.htm](http://www.gn.apc.org/duncan/bat/Health_Committee_Evidence_1.htm). Accessed 2005 Oct 10.
7. . Campbell D, Beelman M, Schelzig E (2000) Paper trail to markets of the East. Guardian, 2 February. Available: <http://www.guardian.co.uk/bat/article/0,2763,191290,00.html>. Accessed 2006 Jan 20.
8. . Leach D. EXISTING BUSINESS STRUCTURE. 21 Aug 1991. British American Tobacco. Bates No. 301131201-210. <http://bat.library.ucsf.edu/data/i/v/n/ivn41a99/ivn41a99.pdf>
9. . Brown WR. State Express Filter King 555 – Product Code 5922, Transit – Hong Kong – Singapore – Bangladesh. 7 Feb 1986. British American Tobacco. Bates No. 301743287-296. <http://bat.library.ucsf.edu/data/r/v/p/rvp01a99/rvp01a99.pdf>
10. . Aitken JM. SECRET, GENERAL MANAGER’S REVIEW. 7 June 1991. British American Tobacco. Bates No. 201823857-872. <http://bat.library.ucsf.edu/data/t/p/l/tpl10a99/tpl10a99.pdf>
11. . Milne I. Volume Analysis BAT Hong Kong and BAT China [Fax to David Potter]. 2 Mar 1993. British American Tobacco. Bates No. 202746431-432. <http://bat.library.ucsf.edu/data/f/x/w/fxw11a99/fxw11a99.pdf>
12. . Lovett N. CHINA DISTRIBUTION & OPERATIONAL STRATEGIES. 1992. British American Tobacco UK & Export. Bates No. 301783358. <http://bat.library.ucsf.edu/pageview?a=view&tid=htu31a99&total=63&page=52>
13. . BAT Hong Kong. CHINA, MARKET ASSUMPTIONS. 1991. Bates No. 301783317-323. <http://bat.library.ucsf.edu/data/h/t/u/htu31a99/htu31a99.pdf>
14. . Gilmore A, McKee M. (2004) Moving east: how the transnational tobacco companies gained entry to the emerging markets of the former Soviet Union. Part I: Establishing cigarette imports. Tobacco Control 13: 143-50.
15. . Herter U. CENTRAL ASIA AND CAUCASUS, Note to the Tobacco Strategy Group. 4 May 1994. British American Tobacco. Bates No. 500036164-366. <http://bat.library.ucsf.edu/data/e/j/z/ejz70a99/ejz70a99.pdf>
16. . Lovett N. CHINA DISTRIBUTION. 1992. British American Tobacco UK & Export. Bates No. 301783334. <http://bat.library.ucsf.edu/pageview?a=view&tid=htu31a99&total=63&page=28&a=view&tid=htu31a99&total=63&page=30>
17. . Coburn R. FILE NOTE HONG KONG MEETING - 22ND SEPTEMBER 1986. 22 Sept 1986. British American Tobacco. Bates No. 301634322-324. Available from Guildford Depository (Accessed 20 May 2003).
18. . Payne J. CHINA, The Past, The Present, The Future [Report Prepared for C.H. Stewart Lockhart]. 29 July 1983. British American Tobacco. Bates No. 301045275. Available from Guildford Depository (Accessed 20 May 2003).
19. . Brockman B. CHINA – BANGLADESH [Letter to David Thorpe]. 20 Oct 1986. British American Tobacco. Bates No. 301634295-296. Available from Guildford Depository (Accessed 20 May 2003).
20. . Thorpe D. NOTES ON FAR EAST VISIT: 24th November – 6th December 1985. 12 Dec 1985. British American Tobacco UK & Export. Bates No. 301766027-029. <http://bat.library.ucsf.edu/data/b/y/p/byp01a99/byp01a99.pdf>
21. . Ng Chye Mong Pte Limited. Shipping invoices [Hong Kong]. 1986. Bates No. 301634345-351. Available from Guildford Depository (Accessed 20 May 2003).
22. . Coburn R. Hong Kong visit - 27th August 1986. 27 Aug 1986. British American Tobacco. Bates No. 301634332-33. Available from Guildford Depository (Accessed 20 May 2003).
23. . Aitken M. re Sino Russian border trade [Letter to Brian Brockman]. 28 Oct 1986. British American Tobacco. Bates No. 301634282-283. Available from Guildford Depository (Accessed 21 May 2003).
24. . BAT US. Tobacco Group, First Half 1987, Quarterly Profit Review [BATUS Board Meeting]. 1987. Bates. No. 201759185 (201759179-203). <http://bat.library.ucsf.edu/pageview?a=view&tid=pgw00a99&total=28&page=9>
25. . Wai Pong. Telex re sales figures. 20 Jan 1987. BAT Hong Kong. Bates No. 301634259-261. Available from Guildford Depository (Accessed 22 May 2003).
26. . Wai Pong. China. March 1989. BAT Hong Kong. Bates No. 301634036-038. Available from Guildford Depository (Accessed 22 May 2003).
27. . Wai Pong. General Managers Monthly Review. January 1989 China. 2 Feb 1989. BAT Hong Kong. Bates No. 301634045-48. Available from Guildford Depository (Accessed 20 May 2003).
28. . Neville MB. NEW PRODUCT CODES. 6 Mar 1990. BAT UK & Export. Bates No. 400034188. <http://bat.library.ucsf.edu/data/t/h/g/thg52a99/thg52a99.pdf>
